# Estrogen Receptor Beta Isoforms Interplay Integrates Nuclear and Extra‐Nuclear Signaling to Modulate Human Granulosa Cell Growth

**DOI:** 10.1096/fj.202602705RR

**Published:** 2026-10-03

**Authors:** Clémentine Marie, Inza‐Noor Baskaran, Raphaël Corre, Anne Mayeur, Michaël Grynberg, Guillaume Chevreux, David L'Hôte, Joëlle Cohen‐Tannoudji, Stéphanie Chauvin

**Affiliations:** ^1^ Unité de Biologie Fonctionnelle et Adaptative, CNRS Université Paris Cité Paris France; ^2^ Service de Médecine de la Reproduction et Préservation de la Fertilité Hôpital Antoine Béclère Clamart France; ^3^ CNRS, Institut Jacques Monod Université Paris Cité Paris France

**Keywords:** cell growth, estradiol, estrogen receptor beta isoforms, extra‐nuclear signaling, human granulosa cells, nuclear signaling, protein interactions, signaling crosstalk

## Abstract

Estradiol (E2) regulates ovarian follicular development and granulosa cell (GC) function primarily through estrogen receptor β (ERβ). Human GCs express several ERβ isoforms, including ERβ1, ERβ2, ERβ4, and ERβ5, but how their relative abundance modulates estrogen signaling remains unclear. Here, we investigated the contribution of nuclear and extra‐nuclear ERβ signaling to human GC growth and function. HGrC1 granulosa cells lacking endogenous estrogen receptors were engineered to stably express individual or combined ERβ isoforms. Functional analyses showed that ERβ1 and ERβ4 inhibited GC proliferation, whereas ERβ2 and ERβ5 promoted cell growth. Co‐expression of all isoforms resulted in an overall inhibitory effect, indicating that GC responses depend on ERβ isoform balance rather than a single receptor. Reporter assays demonstrated that only ERβ1 activated estrogen response element‐dependent transcription, while the other isoforms suppressed E2‐dependent ERα transactivation. In contrast, ERβ2, ERβ4, and ERβ5 activated AP‐1 signaling in a ligand‐independent manner and attenuated ERβ1‐mediated repression, identifying AP‐1 as a central node of ERβ isoform crosstalk. RNA sequencing following selective ERβ1 depletion in primary human granulosa lutein cells revealed that ERβ1 is required for E2 responsiveness and regulates genes involved in membrane potential, ion transport, and cytoskeletal organization. Proteomic and biochemical analyses further identified isoform‐specific signaling networks involving ERK1/2, Akt, and CaMKII pathways. Together, these findings demonstrate that ERβ isoform balance orchestrates estrogen signaling through coordinated nuclear and extra‐nuclear mechanisms controlling GC growth and function.

## Graphical abstract

Working model of ERβ isoform signaling in human granulosa cells. Upon E2 binding, ERβ1 activates ERE‐dependent transcription and represses AP‐1 activity through Jun/Fos interactions. In contrast, ERβ2, ERβ4, and ERβ5 lack E2 binding and ERE activity but promote AP‐1‐dependent transcription and counteract ERβ1 repression, including after phosphorylation by growth factor receptor‐associated kinases. ERβ isoforms also differentially activate CaMKII, ERK1/2, and Akt pathways through isoform‐specific protein interactions, ultimately regulating granulosa cell growth and function.

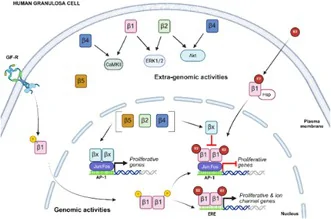

**Abbreviations**
AF‐1: activation function‐1
AF‐2: activation function‐2
Akt: protein kinase B
AP‐1: activator protein‐1
CaMKII: calcium‐calmodulin‐dependent protein kinase II
cAMP: cyclic adenosine 3′,5′‐monophosphate
CTL: control
DBD: DNA binding domain
DEGs: differentially expressed genes
E2: estradiol
ERE: estrogen response element
ERK1/2: extracellular signal‐regulated kinases 44/42
ERα: estrogen receptor alpha
ERβ: estrogen receptor beta
GO: gene ontology
hGC: human granulosa cells
LBD: ligand binding domain
LC‐MS/MS: liquid chromatography coupled to tandem mass spectrometry
MAPK: mitogen‐activated protein kinase
mTOR: mammalian target of rapamycin
PI3‐K: phosphoinositide 3‐kinase
RNA seq: RNA sequencing
RT: room temperature

## 1 Introduction

Estradiol (E2) is a key regulator of a wide range of physiological functions [1]. The tissue‐specific expression and ratio of the two nuclear receptors (ERα and ERβ) determine both E2 sensitivity and type of response. Among its pleiotropic effects, E2 promotes cell proliferation through ERα signaling, whereas activation of ERβ either stimulates proliferation or induces apoptosis depending on the presence of ERα [2]. Both receptors are transcription factors directly binding estrogen‐response elements (EREs) within DNA or tethering to other transcription factors such as the Fos and Jun families (activator protein‐1, AP‐1) [3, 4]. Cooperation between the two activation subdomains located in the N‐terminal region (AF‐1) and the C‐terminal ligand binding domain (LBD, AF‐2) of ERs also contributes to their transcriptional activities [5]. Studies overexpressing ERα and/or ERβ in cancer cell lines have shown that the two receptors share a subset of target genes while also regulating distinct gene sets [6, 7]. Whereas ERα mainly requires ligand binding to modulate transcription, ERβ can regulate up to several hundred genes in the absence of E2 in osteosarcoma cells [8], highlighting the major ligand‐independent activity of ERβ. Ligand‐independent genomic activities occur through phosphorylation of the N‐terminal domain by other signaling pathways, such as those of growth factor receptors [9]. Contrary to ERα, ERβ regulates three classes of genes: Class I genes controlled by unliganded ERβ, Class II genes regulated only in the presence of E2, and Class III genes regulated by unliganded ERβ and further modulated by E2 [8]. EREs are not the predominant DNA‐binding sites of ERβ, as ERβ (particularly in the absence of E2) mostly interacts with genomic regions containing AP‐1 sites [3, 10]. ERβ‐mediated transactivation at the AP‐1 sites is further enhanced by the co‐regulator TIP60, which interacts with the D domain (located between the DNA binding domain, DBD, and the LBD) of ERβ [11]. However, in an E2‐dominated environment, ERβ may shift toward ERE‐mediated transcription [12]. Because ERα and ERβ often coexist within the same cells, various homo‐ or heterodimers can form on DNA [13, 14], both in the absence and presence of E2 [15, 16]. At low E2 concentrations, ERβ acts as an inhibitor of ERα‐dependent transcription, thereby reducing cell growth [17]. In addition to their genomic effects, ERβ and ERα also directly activate extra‐nuclear signaling pathways, including phosphoinositide 3‐kinase (PI3K), cyclic adenosine 3′,5′‐monophosphate (cAMP), and mitogen activated protein kinases (MAPK) cascades, thereby modulating cellular functions and indirectly affecting gene expression [5, 18, 19, 20]. Thus, E2 signaling is highly complex and is still not fully understood.

Five human‐specific ERβ isoforms (ERβ1, ERβ2, ERβ3, ERβ4, and ERβ5) have been identified, adding further complexity to E2 signaling [5]. All isoforms, except ERβ3, which is testis‐specific, are differently expressed in E2‐responsive tissues, where at least two isoforms can coexist [21, 22]. Their functional properties remain poorly characterized [5]. The effects of ERβ1 correspond to those attributed to ERβ in earlier studies, that is, anti‐proliferative and pro‐apoptotic effects [23, 24, 25]. ERβ2 and ERβ5 expression have been reported to be aberrantly altered in various cancer types. Specifically, ERβ2 is upregulated in chronic lymphocytic leukemia (in both lymphoid and myeloid cells) and non‐small cell lung cancer (including squamous cell carcinoma and adenocarcinoma), while both ERβ2 and ERβ5 expression is increased in serous ovarian cancer, as well as in breast and prostate cancers (across epithelial, stromal, endothelial, and immune cells) [26, 27, 28, 29, 30, 31, 32, 33, 34, 35]. Notably, the expression levels of these two isoforms have been shown to correlate with clinical outcomes [29, 30, 34].

ERβ isoforms differ in their C‐terminal regions because of specific alternative splicing of the last exon, exon 8, encoding a part of the LBD [5]. Although they retain DNA‐binding capacity through the conserved DBD [36], all isoforms, except ERβ1, have lost their ability to bind E2 [37]. In neuronal cells, all ERβ isoforms modestly activate basal ERE‐mediated transcription while repressing basal AP‐1‐dependent transcription [36]. ERβ2, ERβ4, and ERβ5 (thereafter referred to as ERβ2‐4‐5) can modulate ERβ1 or ERα activities, enhancing ERβ1‐mediated responses or repressing ERα‐dependent transcription at ERE sites in COS‐7/1 cells [38, 39]. Several isoforms were shown to also act through non‐genomic mechanisms. For example, ERβ5 is reported to activate p44/42 MAPK (ERK1/2), protein kinase B (Akt) [40], mammalian target of rapamycin (mTOR) [25], or Focal adhesion kinase (FAK)/c‐Src signaling pathways in different ovarian cancer lines [41]. ERβ2 regulates interleukin‐12 receptor β2 expression through p38 MAPK signaling in non‐small cell lung cancer lines [42].

In premenopausal females, E2 is produced mainly by the ovary, particularly by follicular granulosa cells (GCs), and plays a central role in fertility by regulating follicle maturation and ovulation [43]. ERβ1 and ERβ2‐4‐5 mRNA have been detected in human ovaries [22, 37], and we recently demonstrated that mature human GCs co‐express low levels of ERα and high levels of mRNA encoding ERβ isoforms [44]. Although ERβ1 and ERβ2‐4‐5 are co‐expressed in preovulatory follicles, the respective biological contribution of each ERβ isoform to GC function remains unknown.

In this study, we provide the first characterization of the combined roles of ERβ isoforms in GC growth by performing an in‐depth analysis of the transcriptional activities of ERβ2‐4‐5 when co‐expressed with ERα or ERβ1 in a human GC line (HGrC1 cells). Using an integrative transcriptomic and proteomic approach, we further identified the specific transcriptomic signature of ERβ1 following E2 activation in primary human GCs and demonstrate that ERβ isoforms may mediate distinct biological functions through multiple nuclear and extra‐nuclear signaling mechanisms, including kinase activation and/or scaffold protein functions. Finally, we highlight the importance of the balance between ERβ isoform expression levels in determining signaling pathway outcomes that ultimately lead to differential cellular growth responses.

## 2 Materials and Methods

### 2.1 Study Participant Details

Five women undergoing in vitro fertilization treatment for infertility due to tubal or male factors at Antoine Béclère Hospital (Clamart, France) were included in this study. Patients underwent controlled ovarian stimulation using clinically established protocols, including GnRH antagonist, long GnRH agonist, and progestin‐primed ovarian stimulation protocols. Follicular fluid was collected by transvaginal ultrasound‐guided aspiration at oocyte retrieval, approximately 36 h after final oocyte maturation triggering with recombinant human chorionic gonadotropin (hCG). Granulosa cells were subsequently isolated from the follicular fluid. Informed and written consent was signed by each participant at the time of recruitment. The study was conducted in accordance with the Declaration of Helsinki for Medical Research Involving Human Subjects (2013 revision). Ethical approval was granted by the institutional review board (IRB Blefco) under registration number “2021‐1.”

### 2.2 Cell Lines and Primary Cell Culture

We used two human GC models with distinct characteristics and limitations: the HGrC1 cell line and primary human GCs obtained following in vitro fertilization (IVF) protocols. HGrC1 cells originate from small, growing follicles and express minute levels of ERα and ERβ isoforms, allowing us to generate cells with controlled ERβ isoform expression. The main limitation of the widely used IVF‐derived GCs is that they are undergoing luteinisation. However, these cells retain endocrine characteristics of differentiated GCs and were therefore used to investigate E2/ERβ‐regulated genomic targets.

#### 2.2.1 Cell Culture

HGrC1 cells [45] (RRID:CVCL_KB28), were cultured in Dulbecco's Modified Eagle Medium/Nutrient Mixture F‐12 Ham (DMEM/F12, 1:1; Thermo Fisher Scientific, #12634010) supplemented with 10% fetal calf serum (FCS; Thermo Fisher Scientific, #10371029) and 10,000 U/mL penicillin–streptomycin (PS; Thermo Fisher Scientific, #15140122), referred to hereafter as growth medium. Cells were maintained at 37°C in a humidified atmosphere containing 5% CO_2_. HEK293T and HCT116 cells, obtained from the American Type Culture Collection (ATCC; CRL‐3216 and CCL‐247EMT, respectively), were cultured in DMEM (Thermo Fisher Scientific, #32430100) supplemented with 10% FCS and PS under the same incubation conditions.

#### 2.2.2 Primary Cultured Granulosa Lutein Cells

After oocyte retrieval, granulosa lutein cells (hGCs) were purified from the follicular fluid. Briefly, follicular fluid was centrifuged through a one‐step Percoll gradient (v/v, Dulbecco's phosphate‐buffered saline [DPBS]/Percoll; Thermo Fisher Scientific, #11500744) at 4000 × *g* for 15 min to remove red blood cells. hGCs were collected at the interface, washed with DPBS, resuspended in growth medium, and cultured at 37°C in a humidified incubator with 5% CO_2_.

#### 2.2.3 Cell Treatments

Twenty‐four hours after seeding in 12‐well plates, hGCs were further cultured for 24 h in phenol red‐free DMEM/F12 medium (Thermo Fisher Scientific, #21041025) supplemented with 10% charcoal‐dextran‐stripped FCS and PS (hereafter referred to as treatment medium). Cells were then treated for an additional 24 h with either 10 nM E2 (Sigma, #E1132), a concentration representative of the intrafollicular concentration of dominant follicles of normally cycling fertile women [46, 47, 48], or an equivalent volume of ethanol (vehicle control, V).

### 2.3 Methods Details

#### 2.3.1 Reagents

The primary antibodies used in this study are anti‐Phospho‐AKT (Ser473) (Cell Signaling, #4060), anti‐Phospho‐ERK1/2 (Thr202/Tyr204) (Cell Signaling, #9101), anti‐Phospho‐CaMKIIα (Thr286) (Santa Cruz, sc‐13 082), anti‐GAPDH (Sigma, G8795), anti‐AKT (Cell Signaling, #9272), anti‐ERK1/2 (Cell Signaling, #91025), anti‐Flag M2 (Sigma, F1804), and anti‐BrdU (Abcam, ab152095). Secondary antibodies included Alexa Fluor 488‐conjugated anti‐rabbit (Molecular Probes, Thermo Scientific, #A32790) and Alexa Fluor 568‐conjugated anti‐mouse (Molecular Probes, Thermo Scientific, #A11019), as well as horseradish peroxidase (HRP)‐conjugated goat anti‐rabbit and goat anti‐mouse antibodies (Thermo Fisher Scientific, #31460 and #31430, respectively). The Western blot stripping buffer used was Restore Western Blot Stripping Buffer (Thermo Scientific, #21059). For immunoprecipitation, EZview Red anti‐FLAG M2 agarose beads (Sigma, #F2426) were employed. Antibiotics used for selection of stable cell lines included puromycin (Invivogen, ant‐pr‐1), hygromycin B Gold (Invivogen, ant‐hg‐1), G418 (Invivogen, ant‐gn‐1), and zeocin (Invivogen, ant‐zn‐05). The MTT reagent for cell proliferation assays was MTT formazan (Sigma, #M2003). RNA extraction was performed using TRIzol Reagent (Thermo Fisher Scientific, #A33248), cDNA synthesis with SuperScript II reverse transcriptase (Thermo Fisher Scientific, #18064014), and quantitative PCR amplification with SYBR Green PCR Master Mix (Roche, FSUSGMMRO). RNA purification was conducted using the RNeasy Micro Kit (Qiagen, #74004).

#### 2.3.2 Expression Vectors

Flag‐ERβ1, Flag‐ERβ2, Flag‐ERβ4, and Flag‐ERβ5 vector constructions have been described previously [44]. The Flag‐ERα expression vector was kindly provided by Dr. F. Lenfant (I2MC, Toulouse, France). We generated a Flag‐Cherry construct (hereafter referred to as Flag‐control) by PCR amplification from pmCherry‐C1, which encodes a monomeric derivative of DsRed fluorescent protein of about 26.7 kDa (Clontech, #632524), following previously reported methods [44], using the forward primer (Eurofins Genomics) 5′‐**GGGGGG** *GCGGCCGC*GATGGTGAGCAAGGGCGAGG‐3′ and the reverse primer 5′‐**CCCCCC** *GGATCCTCA*CTTGTACAGCTCGTCC‐3′ (*NotI* and *BamHI* restriction sites in italics). The estrogen‐responsive reporter plasmid and the AP1‐responsive reporter plasmid contain three copies of vitellogenin ERE sequence and six copies of the AP‐1‐sequence (TGACTCA) upstream of luciferase (ERE‐Luc and AP‐1‐Luc, respectively) (Addgene, Watertown, MA 02472, USA, #11354, and Promega, Charbonnières‐les‐bains, France, #11783), respectively. The plasmid pRL‐TK, carrying the Renilla luciferase gene under the control of the TK promoter (Promega, #E2231) was used as an internal control of transfection efficiency in reporter assays.

#### 2.3.3 Construction of Stable Expression Vectors

Constructs were generated using the MXS‐chaining strategy developed by Sladitschek and Neveu [49]. This method enables modular vector assembly through iterative cloning. The SMX_DEST vector and various building cassettes were from Addgene (MXS_Chaining Kit #1000000064). We used vectors encoding the reverse tetracycline transactivator (rtTA3) under the control of the phosphoglycerate kinase (PGK) promoter, and a bidirectional tetracycline‐inducible promoter (BiTet) driving expression of both a fluorescent reporter gene (either mEGFP (488/507 nm), mCherry (587/610 nm), TagBFP (399/456 nm), or Citrine (516/529 nm)) and a given *ESR2* isoform (ERβ1/2/4 or 5). The vectors also contained antibiotic resistance markers (puromycin (Puro^R^), hygromycin (Hygro^R^), neomycin (Neo^R^), or zeocin (Zeo^R^)) under the control of the PGK promoter, along with bovine growth hormone polyadenylation signals (bGHpA) downstream of each coding sequence.

The MXS cloning strategy relies on the compatible cohesive ends of *SalI* and *XhoI*, with *MluI* as the direction‐defining enzyme. In the MXS standard, each module is flanked by *MluI* and *XhoI* sites at its 5′‐end and a *SalI* site at its 3′‐end. To assemble two modules in the desired order, the 5′‐module is isolated by a *MluI*/*SalI* double digest and ligated to a *MluI*/*XhoI*‐linearized vector containing the 3′‐module. The *SalI* and *XhoI* overhangs are compatible and produce a translatable scar upon ligation.

The cDNAs encoding ERβ1 (*ESR2v1*), ERβ2 (*ESR2v2*), ERβ4 (*ESR2v4*), and ERβ5 (*ESR2v5*) with flanking *XhoI* and *MluI* sites were amplified by PCR (as previously described [44]) from pcDNA_4_ plasmids containing *ESR2* variants, using primers listed in Table S1. The identity and integrity of all newly cloned modules were confirmed by Sanger sequencing using the forward primer 5′‐TTACCGCCTTTGAGTGAG‐3′ and reverse primer 5′‐TTGTCTCATGAGCGGATAC‐3′, which bind to conserved regions in the minimal backbone flanking each MXS module. Given that the final vector sizes ranged from 7728 to 8478 bp (depending on the ERβ isoform), additional primers were used for sequence verification: one pair binding within the BiTet sequence (forward: 5′‐GTGAAAGTCGAGTTTACCACTCC‐3′; reverse: 5′‐CTGATAGGGAGTGGTAAACTCG‐3′), and one pair flanking the ESR2 coding sequence (forward: 5′‐TTACCGCCTTTGAGTGAG‐3′; reverse: 5′‐TTGTCTCATGAGCGGATAC‐3′). As a result, we generated four pairs of expression and corresponding control vectors for stable transfection into HGrC1 cells: *Vector 1a (ESR2v1)*: MXS‐bGHpA::ESR2v1::BiTet::EGFP::bGHpA::PGK::PuroR::bGHpA::PGK::rtTA3::bGHpA; *Vector 1b (Control for ESR2v1)*: MXS‐BiTet::EGFP::bGHpA::PGK::PuroR::bGHpA::PGK::rtTA3::bGHpA; *Vector 2a (ESR2v2)*: MXS‐bGHpA::ESR2v2::BiTet::mCherry::bGHpA::PGK::HygroR::bGHpA::PGK::rtTA3::bGHpA; *Vector 2b (Control for ESR2v2)*: MXS‐BiTet::mCherry::bGHpA::PGK::HygroR::bGHpA::PGK::rtTA3::bGHpA; *Vector 3a (ESR2v4)*: MXS‐bGHpA::ESR2v4::BiTet::TagBFP::bGHpA::PGK::NeoR::bGHpA::PGK::rtTA3::bGHpA; *Vector 3b (Control for ESR2v4)*: MXS‐BiTet::TagBFP::bGHpA::PGK::NeoR::bGHpA::PGK::rtTA3::bGHpA; *Vector 4a (ESR2v5)*: MXS‐bGHpA::ESR2v5::BiTet::Citrine::bGHpA::PGK::ZeoR::bGHpA::PGK::rtTA3::bGHpA; *Vector 4b (Control for ESR2v5)*: MXS‐BiTet::Citrine::bGHpA::PGK::ZeoR::bGHpA::PGK::rtTA3::bGHpA.

#### 2.3.4 Construction of Lentiviral Vectors

Lentiviral vectors encoding predesigned short hairpin RNAs (shRNAs) targeting *ESR2* variant 1 (ERβ1) (designated B1) or a non‐targeting scrambled control (S) were constructed using the pLKO.1‐puro backbone (Addgene, #10878), which confers puromycin resistance for selection. shRNA sequences were designed based on published protocols [50, 51], and a BLAST search (https://www.ncbi.nlm.nih.gov/BLAST) was performed to ensure specificity and exclude potential off‐target homology. Sense and antisense 5′‐phosphorylated oligonucleotides were designed to anneal into a double‐stranded DNA fragment containing the shRNA sequence, flanked by *AgeI* and *EcoRI* cohesive ends for cloning. The sequences of the shRNAs used are provided in Table S2. Oligonucleotides were ligated into the digested pLKO.1‐puro vector and transformed into *E. coli* Stbl3 cells (Invitrogen, #C7373‐03). Plasmid DNA was prepared using an endotoxin‐free purification kit (Macherey‐Nagel, Ref. 740548) and sequence‐verified prior to lentiviral production. Lentiviruses were produced by co‐transfecting HEK293T cells with the shRNA‐expressing vector, the packaging plasmid psPAX2, and the envelope plasmid pMD2.G using the calcium phosphate transfection method. Viral supernatants were harvested 36 h post‐transfection and concentrated 100‐ to 1000‐fold by ultracentrifugation, as previously described [52]. Viral titers were evaluated by transduction of HCT116 cells followed by puromycin selection. Viral particles were aliquoted and stored at −80°C for long‐term use. Viral production was performed by the VVTG platform (Structure Fédérative de Recherche Necker, UMS 24/UMS 3633, Faculté de Santé, Université Paris Cité, France).

#### 2.3.5 RNAseq Library Preparation and Sequencing of Primary hGC Cultured Samples

Extracted RNA samples underwent quality control (QC) using the Agilent Bioanalyzer 2100 and were quantified with a Qubit Fluorometer (Thermo Fisher Scientific). cDNA library preparation and RNA sequencing were carried out by Novogene (Cambridge, UK). Libraries were prepared from 200 ng of input RNA and PCR‐amplified indexed libraries were sequenced on the Illumina NovaSeq 6000 platform (paired‐end 150 bp reads), generating approximately 40 million reads per sample.

#### 2.3.6 High‐Throughput RNA Sequencing Data Processing

Raw RNA‐seq FASTQ files were processed for quality control and adaptor trimming using *fastp* (RRID: SCR_016962). Cleaned reads were aligned to the human reference genome (hg38) using HISAT2 version 2.0.5 [53] within the Illumina BaseSpace RNA‐seq Alignment application. These preprocessing and alignment steps were performed by Novogene (UK). Following alignment, gene‐level read counts were generated from BAM files using a reference gene annotation (GENCODE, hg38), producing raw integer count matrices. These raw counts were used exclusively as input for downstream statistical analysis. Transcript abundance was additionally estimated as FPKM (Fragments Per Kilobase of transcript per Million mapped reads) using Cufflinks for descriptive purposes only and was not used for differential expression testing. Differential expression analysis comparing control (CT) and E2‐treated patient samples (E2) was performed using DESeq2 (v1.40.2), which models raw count data using a negative binomial distribution and applies internal normalization for library size. Nominal *p*‐values derived from DESeq2 were used for visualization in volcano plots, whereas adjusted *p*‐values (Benjamini–Hochberg false discovery rate) were used to define significantly differentially expressed genes and to generate gene lists. Genes with log_2_(fold change) ≥ 1 and adjusted *p*‐value ≤ 0.05 were considered significant. Principal component analysis (PCA) was conducted on log‐transformed counts per million (log‐CPM) derived from raw count data using the FactoMineR package to assess sample clustering and variability between experimental groups. The RNA‐seq dataset has been deposited at ArrayExpress (EMBL' European Bioinformatics Institute) as accession number E‐MTAB‐16651 and are publicly available as of the date of publication.

#### 2.3.7 Viral Infection and E2 Treatment of Primary Cultures of Granulosa Cells

Primary cultured hGCs were seeded in 12‐well plates at 0.6 × 10^6^ cells per well in growing medium and maintained at 37°C for 24 h. Cells were then transduced with viral particles at a multiplicity of infection (MOI) of 90 viral particles per cell (optimized titer) in 250 μL of DMEM/F12 medium without phenol red. Three hours post‐infection, treatment medium containing 2 μg/mL puromycin was added to ensure selection of successfully transduced cells. Twenty‐four hours after transduction, cells were treated for an additional 24 h with either vehicle or 10 nM E2 in fresh treatment medium containing puromycin. Total RNA was extracted from each condition using the RNeasy Micro Kit (Qiagen), from five independent patient‐derived hGC cultures. An aliquot of 0.5 μg RNA per sample was used for assessing knockdown efficiency and target specificity by quantitative PCR (qPCR), targeting the specific exon site. The remaining RNA was used for RNA sequencing (RNA‐seq) analysis.

#### 2.3.8 Establishment of Stable HGrC1 Cell Lines

A total of 5 × 10^6^ cells was electroporated with linearized plasmids (vectors 1a, 1b, 2a, 2b, 3a, 3b, 4a, or 4b) using the Neon Transfection System (Invitrogen) according to the manufacturer's protocol (three pulses at 1600 mV for 10 ms). Stable cell lines expressing each *ESR2* isoform or the corresponding control were generated by antibiotic selection using the following concentrations: puromycin (1 μg/mL) for vectors 1a (*ESR2v1*) and 1b (control); hygromycin B Gold (200 μg/mL) for vectors 2a (*ESR2v2*) and 2b (control); G418 (200 μg/mL) for vectors 3a (*ESR2v4*) and 3b (control); and zeocin (400 μg/mL) for vectors 4a (*ESR2v5*) and 4b (control). Once colonies became visible (after ~4 weeks), individual clones (~100 cells) were isolated by micro‐trypsinization in cloning cylinders under a fluorescent microscope, allowing visualization of fluorescently labeled cells (mEGFP, mCherry, TagBFP, or Citrine for ERβ1, ERβ2, ERβ4, and ERβ5‐expressing clones and their controls, respectively). Following limiting dilution, clones were expanded and analyzed by RT‐qPCR using the primers listed in Table S3.

To generate stable cell lines expressing all four ERβ isoforms (4β) and the corresponding control line (CTL_4β_), the β1‐expressing line was stably transfected with vector set 2a, while the β1‐control line received vector set 2b. Clones were selected in medium containing both puromycin and hygromycin B, and screened for *ESR2v1* and *ESR2v2* transgene expression by RT‐qPCR. Two additional rounds of stable transfection were performed using the same strategy to obtain HGrC1 cell lines expressing all four ERβ isoforms, along with their respective control lines. This order of transgene integration proved to be the most efficient. At least two clones per condition were selected for further analysis based on comparable mRNA expression levels of the estrogen‐responsive gene *GREB1* involved in cell growth, and the GCs marker Forkhead Box L2 *FOXL2*, relative to the parental HGrC1 cells. The following cell lines used in this study were:

– β1#4 HGrC1 line and its control (CTL β1#1 HGrC1 line).
– β2 #1 HGrC1 line and its control (CTL β2#2 HGrC1 line).
– β4 #4 HGrC1 line and its control (CTL β4#1 HGrC1 line).
– β5 #1 HGrC1 line and its control (CTL β5#2 HGrC1 line).
– 4β #7 HGrC1 line and its control (CTL 4β#1 HGrC1 line).

#### 2.3.9 Growth Assay (MTT)

Stable or transiently transfected HGrC1 cells were seeded at 4 × 10^4^ cells per well in 24‐well plates in growing medium. At specified time points (24, 48, 72, and 96 h), MTT reagent (5 mg/mL in PBS) was added to wells at a final concentration of 0.9 mg/mL. Cells were incubated for 2 h at 37°C and 5% CO_2_. After removal of supernatant, cells were lysed with 200 μL DMSO for 10 min to dissolve formazan crystals. Absorbance at 575 nm was measured using a FlexStation 3 ELISA plate reader. Fold change in growth was calculated as the ratio of absorbance at 48, 72, or 96 h to that at 24 h post‐transfection.

#### 2.3.10 RNA Extraction and mRNA Quantification

Total cellular RNA was extracted using TRIzol Reagent according to the manufacturer's protocol. Two micrograms of RNA were reverse‐transcribed into cDNA using SuperScript II reverse transcriptase and random primers, according to the manufacturer's instructions. Control reactions were performed without RNA polymerase addition. Quantitative PCR (qPCR) was performed on a LightCycler 480 II System (Roche) using SYBR Green Master Mix. All samples were run in duplicate and normalized to *GAPDH* and *ACTB* expression as internal controls. Experiments were independently repeated at least three times. Primer sequences are provided in Table S3. Relative gene expression levels were calculated as previously described [44]. qPCR efficiency exceeded 90% for all genes analyzed (Table S3).

Semi‐quantitative PCR was also performed to assess the expression of each ERβ isoform in individual clones, as well as the 4β clone, for which all isoforms cannot be accurately quantified by conventional qPCR. Common primers targeting ERβ1, ERβ2, ERβ4, and ERβ5 were designed in the 3′‐end of the coding sequence and the 5′‐end of bovine growth hormone polyadenylation signal: forward, 5′‐CTCCTGATGCTCCTGTCCCA‐3′; reverse, 5′‐GGCACAGTCATTCATTCACTCGA‐3′. Optimal cycle numbers within the exponential phase (cycles 21.8–23.6 depending on the isoform) were determined beforehand by qPCR using the same RNA samples. Conventional RT‐PCR was performed using GoTaq DNA polymerase and random primers, according to the manufacturer's instructions (#M3001; Promega). Reactions were run in a thermal cycler (Mastercycler Nexus X2 Eppendorf; Dutscher, Bernolsheim, France) with the following conditions: PCR activation for 4 min at 95°C, followed by 35 cycles of 94°C for 30 s, 60°C for 30 s and 72°C for 30 s, and a final extension at 72°C for 10 min. Aliquots (10 μL) were collected at 20, 25, and 30 cycles. PCR products were separated on a 5% agarose gel, stained with 0.5 μg/mL ethidium bromide (Sigma #E1510) in 1xTAE (Sigma #6549) and visualized under UV light (Biorad GelDoc XR+ imaging system). Expected amplicon sizes were 246 bp (ERβ1), 141 bp (ERβ2), 91 bp (ERβ4), and 71 bp (ERβ5). Band intensities were quantified using Image J.

#### 2.3.11 Transient Transfection and Proliferation Assay Using BrdU Incorporation and Immunofluorescence

HGrC1 cells were transiently transfected with Flag‐tagged constructs encoding mCherry (CTL), ERβ1, ERβ2, ERβ4, or ERβ5 using nucleofection with the Amaxa Cell Line Optimization Nucleofector Kit (Lonza, Cologne, Germany) according to previously described protocols [44]. Transfected HGrC1 cells were seeded at 2 × 10^4^ cells per well in 8‐well Nunc Lab‐Tek chambers containing growing medium and incubated at 37°C with 5% CO_2_ for 48 h. Cells were then incubated with 10 μM BrdU (5‐bromo‐2′‐deoxyuridine; Sigma, **#**B9285) for 2 h and fixed with 4% (w/v) paraformaldehyde for 15 min at room temperature (RT). After three washes with PBS, cells were permeabilized with 0.1% Triton X‐100 in PBS containing 1% (w/v) bovine serum albumin (BSA) for 6 min, followed by incubation in 2N HCl for 20 min. After three additional PBS washes, cells were blocked with 3% BSA in PBS for 1 h at RT, then incubated overnight at 4°C with mouse anti‐Flag M2 antibody (1:250) and rabbit anti‐BrdU antibody (1:200). Following five washes with 0.1% Tween‐20 in PBS, cells were incubated for 1 h at RT with Alexa Fluor 488‐conjugated anti‐rabbit (1:2000) and Alexa Fluor 568‐conjugated anti‐mouse secondary antibodies (1:2000). Cells were washed five times with 0.1% Tween‐20 in PBS and nuclei counterstained with 300 nM DAPI (Sigma, #MBD0015). Samples were mounted in ProLong Gold antifade reagent (Thermo Fisher Scientific, #P36930). Fluorescent images were acquired using a Nikon Eclipse 90i microscope equipped with an AxioCam HRc (Zeiss). Multiple fields were imaged at ×20 magnification. The proliferation rate was quantified as the percentage of BrdU‐positive cells among Flag‐positive cells, analyzing at least 100 transfected cells per condition. Each transfection was performed in a minimum of six independent replicates.

#### 2.3.12 Transactivation Assays

The Firefly luciferase reporter construct containing three copies of ERE (3xERE‐TATA‐luc, Addgene #11354, Watertown, MA 02472, USA) or six copies of an AP‐1‐response element (AP‐1 pGL4.44 [luc2P/AP1 RE/Hygro], Promega E4111) were used in this study. The control plasmid pRL‐TK, carrying the Renilla luciferase gene under the control of the TK promoter (Promega E2241) was co‐transfected to correct for variations in transfection efficiency. The reporter (ERE‐ or AP‐1 sequences, pGL2 or pGL4.44 backbones at 125 ng/well) and the control (Renilla at 20 ng/well) plasmids were transfected using Lipofectamine 2000 reagent (Life Technologies) in OptiMEM medium (Life Technologies) according to the manufacturer's protocol for 6 h. Then, the medium was replaced with treatment medium for 24 h, and cells were treated with vehicle or E2 for another 24 h, as previously described [44]. Cell lysates were then prepared and assayed using the Dual‐Luciferase Reporter Assay System (Promega, #E1910). Luminescence was measured on a Lumat LB 9507 luminometer equipped with an automatic dual injector system (Berthold Technologies, Germany). Firefly luciferase activity was normalized to Renilla luciferase activity, and results are expressed as the ratio of Firefly/Renilla relative light units (RLU). Each experimental condition was performed in six technical replicates, and all assays were repeated in at least four independent experiments.

#### 2.3.13 Protein Extraction and Western Blot

Cellular proteins were extracted using a modified RIPA lysis buffer containing 150 mM NaCl, 50 mM Tris (pH 8), 1% Triton X‐100, 0.1% SDS, 50 mM NaF, 1 mM EDTA, 1 mM EGTA, and 0.5% sodium deoxycholate, supplemented with a protease inhibitor cocktail (Roche, #04693116001). Protein samples were separated on 10% SDS‐PAGE gels and transferred onto nitrocellulose membranes. Membranes were blocked with 5% non‐fat milk in 0.01% Tween‐20 in TBS (TBS‐T) for 1 h at RT and then incubated overnight at 4°C with primary antibodies diluted in 5% non‐fat milk in TBS‐T: Flag M2 (1:1000), P‐ERK1/2 (1:1000), P‐Akt (1:1000), P‐CaMKII (1:300), GAPDH (1:10000), ERK1/2 (1:1000), or Akt (1:1000). Following extensive washes, membranes were incubated with horseradish peroxidase (HRP)‐conjugated secondary antibodies (1:10000 dilution in TBS‐T with 5% milk) for 2 h at RT, then washed again. Immunodetection was performed using the Pierce ECL Plus Western Blotting substrate (Thermo Scientific, #34580). Protein signals were quantified by densitometry using a CCD camera system (LAS4000, FUJI) and normalized to GAPDH (for CaMKII blot) or to total AKT or total ERK1/2 signals after membrane stripping with Restore Western Blot Stripping Buffer (Thermo Scientific). Image analysis was conducted with ImageJ software (NIH). All western blots shown are representative of at least three independent experiments. Uncropped blot images are provided in Figure S1.

#### 2.3.14 Immunoprecipitation of Flag‐Tagged ERβ Isoforms for Mass Spectrometry

Fifteen million (15 × 10^6^) HGrC1 cells were electroporated with plasmids encoding Flag‐vector, Flag‐ERβ1, Flag‐ERβ2, Flag‐ERβ4, or Flag‐ERβ5 isoforms using a Neon Transfection System (three pulses at 1600 mV for 10 ms). Cells were seeded into two separate 100 mm dishes containing growing medium, except for ERβ1‐transfected cells, which were seeded in treatment medium. Twenty‐four hours after transfection, ERβ1‐expressing cells were treated with vehicle or 10 nM E2 for an additional 24 h. For all conditions, proteins were extracted using a modified RIPA lysis buffer (150 mM NaCl, 50 mM Tris pH 8, 1% Triton X‐100, 1 mM EDTA, 1 mM EGTA, 50 mM NaF, 1 mM Na_3_VO_4_, and 1X protease inhibitor cocktail). Cells were mechanically lysed by passing the lysate six times through a 26½ gauge needle, followed by sonication (8 × 5 s at 20 kHz). Whole cell lysates were then rotated for 2 h at 4°C before centrifugation at 16 000 × *g* for 30 min at 4°C. The resulting lysate was incubated overnight at 4°C with EZview Red anti‐FLAG M2 agarose beads under rotation. After incubation, beads were washed three times with TBS to remove non‐specific proteins. The agarose resin, now coupled with Flag‐tagged protein complexes, was collected and prepared for subsequent mass spectrometry analysis. An aliquot of each sample was kept and analyzed by western‐blotting to assess presence and integrity of the bait protein Flag‐ERβ in ERβ transfected cells compared to control Flag sample (Figure S2). Beads from pulldown experiments were incubated overnight at 37°C with 20 μL of 50 mM NH_4_HCO_3_ buffer (Sigma) containing 1 μg of sequencing‐grade trypsin (Promega). The digested peptides were loaded and desalted on evotips (Evosep, Odense, Denmark) according to manufacturer's procedure.

#### 2.3.15 Liquid Chromatography Coupled to Tandem Mass Spectrometry (LC–MS/MS) Acquisition and Analysis

Samples were analyzed on a timsTOF Pro 2 mass spectrometer (Bruker Daltonics, Bremen, Germany) coupled to an Evosep one system (Evosep, Odense, Denmark) operating with the 30SPD method developed by the manufacturer. Briefly, the method is based on a 44 min gradient and a total cycle time of 48 min with a C18 analytical column (0.15 × 150 mm, 1.9 μm beads, ref. EV‐1106) equilibrated at 40°C and operating at a flow rate of 500 nL/min. H_2_O/0.1% MS grade formic acid (FA, ThermoFisher Scientific) was used as solvent A, and acetonitrile (can)/0.1% FA as solvent B. The timsTOF Pro 2 was operated in PASEF mode over a 1.3 s cycle time. Mass spectra for MS and MS/MS scans were recorded between 100 and 1700 *m*/*z*. Ion mobility was set to 0.75–1.25 V·s/cm^2^ over a ramp time of 180 msec. Data‐dependent acquisition was performed using 6 PASEF MS/MS scans per cycle with a near 100% duty cycle. Low *m*/*z* and singly charged ions were excluded from PASEF precursor selection by applying a filter in the *m*/*z* and ion mobility space. The dynamic exclusion was activated and set to 0.8 min, a target value of 16 000 was specified with an intensity threshold of 1000. Collisional energy was ramped stepwise as a function of ion mobility. MS raw files were processed using PEAKS Online 12 (build 2.0, Bioinformatics Solutions). Data were searched against the *Homo sapiens* SwissProt database (release 2024_01, total entry 20 429). Parent mass tolerance was set to 20 ppm, with fragment mass tolerance at 0.05 Da. Specific tryptic cleavage was selected and a maximum of 2 missed cleavages was authorized. For identification, the following post‐translational modifications were included: oxidation (M), acetylation (Protein N‐term) and deamidation (NQ) as variables and half of a disulfide bridge (C) as fixed. Identifications were filtered based on a 1% FDR (false discovery rate) threshold at both peptide and protein group levels. Label free quantification was performed using the PEAKS Online 12 quantification module, allowing a mass tolerance of 5 ppm, a CCS error tolerance of 0.05 and 0.25‐min retention time shift tolerance for match between runs. Protein abundance was inferred using the top N peptide method and TIC was used for normalization.

Approximately 5000 proteins were initially identified. To ensure high‐confidence interaction data, stringent filtering criteria were applied: ≥ 4 unique peptides for quantification, an average abundance (area) > 500 (for reference, the ESR2 bait showed an area of 337 902). With these filtering criteria, only 2451 protein entries remained. To filter differential interacting proteins by comparison with the Flag‐control experiment, we calculated a ratio based on the following formula: ratio = Flag‐ERβ area/(Flag‐control area + Flag‐ERβ area) × 100. If a protein has the same abundance in Ctrl versus ERβ condition the calculated ratio would be of 50. If a protein is only present in the ERβ condition, the ratio would be of 100. To remain consistent, we established a ratio of at least 90% to filter out differential interacting proteins, and efficiently recovered NCOR1, VASP, FXR, and HSP90AB1. The mass spectrometry proteomics data have been deposited at ProteomeXchange Consortium via the PRIDE partner repository as dataset identifier PXD073667 and are publicly available as of the date of publication.

### 2.4 Statistical Analysis

Means ± standard error of the mean (SEM) of *n* independent experiments of measurements were analyzed for significant difference between groups by one‐way ANOVA corrected for multiple comparisons using the Tukey statistical test, or by Mann–Whitney *U* tests when comparing cells treated with either vehicle (V) or E2 using GraphPad Prism 9.0 software (GraphPad Software, La Jolla, CA, USA). Significance was set at *p* value ≤ 0.05.

For transcriptomic data, protein signal intensity was converted to a log_2_ scale, and biological replicates were grouped by experimental condition. Volcano plot shows the log_2_(fold change) on the *x* axis and the −log_10_(*p* value) on the *y* axis. The transcriptomic cut‐off values for analysis were a log_2_(fold change) of 1 and an adjusted *p*‐value ≤ 0.05, which were determined using t‐statistics. Each figure consists of a minimum of three independent experiments from multiple biological replicates, except the LC–MS/MS analysis of tagged transfected HGrC1 performed once.

## 3 Results

### 3.1 Combinatorial Actions of ERβ Isoforms Regulate GC Growth

To investigate the individual and combined effects of ERβ isoforms on GC growth, we used HGrC1 cells, the only currently available immortalized human GC line. HGrC1 cells express minute levels of ERα and ERβ isoforms [44], allowing the analysis of specific mechanisms displayed by given expressed isoforms. We generated clonal stable HGrC1 lines expressing a given ERβ isoform (*ESR2* variants v1, v2, v4, or v5), along with a specific fluorescent protein, both under the control of a BiTet‐inducible promoter. Control cell lines expressed only the corresponding fluorescent proteins (Figure S3A). Because HGrC1 cells were originally immortalized using a Tet‐Off system [45], the introduced BiTet promoter is constitutively activated by the endogenously expressed tetracycline‐controlled transactivator (tTA), resulting in continuous expression of the isoforms and their respective fluorescent markers. Transgene expression was confirmed by RT‐qPCR in single ERβ isoform‐expressing lines (Figure S3B), and results showed similar levels of expression of all isoforms, as previously observed for endogenous expression in human GCs. Due to the lack of reliable antibodies [54], ERβ isoforms' protein levels could not be assessed. To exclude any potential effects of the antibiotic‐based stable transgene integration system on cell growth, we compared the growth rates of parental HGrC1 cells with those of the four stable control lines (Figure S4A). All five cell types displayed comparable growth rates, indicating that the selection procedure did not significantly affect cell growth.

As shown in Figure 1A, after 48 h of culture in basal conditions, HGrC1 cells expressing ERβ1 or ERβ4 exhibited a significant reduction in cell growth (~0.28‐fold and ~0.32‐fold, respectively; *p* ≤ 0.01), which persisted throughout the 96‐h culture period. In contrast, ERβ2 expression significantly enhanced cell growth (~1.62‐fold; *p* ≤ 0.001) at 96 h, and ERβ5 expression induced an even earlier increase, starting at 72 h (~1.90‐fold; *p* ≤ 0.05) and reaching ~2.12‐fold (*p* ≤ 0.01) at 96 h. These findings reinforce and extend our previous observations on HGrC1 cells transiently transfected with Flag‐tagged ERβ isoforms [44]. To better mimic the physiological context within ovarian follicles, we generated HGrC1 cell lines stably co‐expressing all four ERβ isoforms through sequential transfections using distinct antibiotic resistance markers. Among the resulting clones, we selected clone 4β#7, hereafter referred to as 4β, which expresses similar levels of each isoform as compared to single‐isoform cell lines (Figures S3B and S4B). As shown in Figure 1B, the 4β‐expressing cell line exhibited a significant reduction in cell growth after 48 h of culture (~0.25‐fold decrease relative to 24 h control; *p* ≤ 0.01), which persisted through 96 h. A comparable decrease in cell growth was also observed in an additional 4β‐expressing clone (clone #22; Figure S4C), further supporting the finding that combined expression of all four ERβ isoforms attenuates HGrC1 cell growth. We next assessed whether the reduced growth was associated with altered expression of proliferation and/or apoptosis‐related genes reported to be regulated by ERβ [24, 55, 56, 57], including *EGR1*, *MYC*, *CTNNB, BAX*, and *BCL2*. To this end, RT‐qPCR analysis was performed in both single‐isoform and 4β‐expressing lines, along with their respective controls, 96 h after seeding. As shown in Figure 1C, expression of ERβ2 and ERβ5 significantly upregulated *EGR1* (~2.57‐fold; *p* ≤ 0.001 and ~1.49‐fold compared to CTL; *p* ≤ 0.05, respectively), *MYC* (~1.39‐fold; *p* ≤ 0.01 and ~1.61‐fold; *p* ≤ 0.001, respectively) and *CTNNB1* (only by β5, ~1.16‐fold; *p* ≤ 0.05). These findings are consistent with the proliferation phenotypes observed following transient expression of tagged forms of ERβ2 or ERβ5 isoform in HGrC1 cells (Figure S5). Conversely, ERβ1 and ERβ4 expression significantly downregulated *CTNNB1* (~0.17‐fold and ~0.37‐fold compared to CTL; *p* ≤ 0.001, respectively), with ERβ1, but not ERβ4, also significantly reducing *MYC* expression (~0.33‐fold; *p* ≤ 0.01). ERβ1 expression did not significantly alter *EGR1* expression, whereas ERβ4 showed a trend toward decreased *EGR1* expression (*p* = 0.053) (Figure 1C). The ratio between *BAX*/*BCL2* mRNA was similar in all β‐expressing clones (Figure 1C), suggesting that ERβ isoforms do not regulate these apoptosis‐related genes in this cellular context.

**FIGURE 1:**
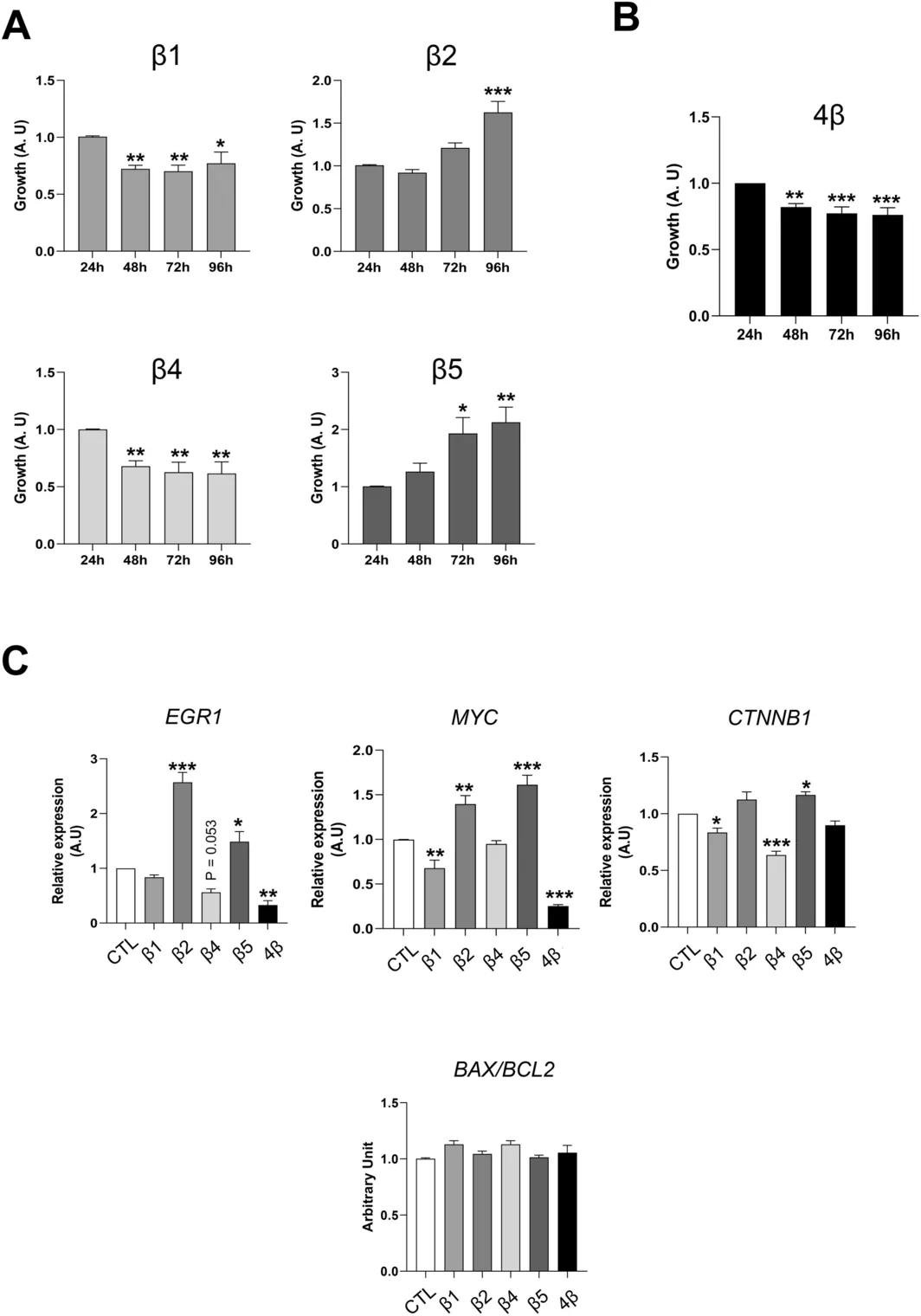
Differential effects of ERβ isoforms on granulosa cell growth. (A) Growth of HGrC1 cells stably expressing ERβ1, ERβ2, ERβ4, or ERβ5, compared with their respective controls, was assessed by MTT assay at 24–96 h after seeding. Results are expressed as fold change in OD_575_ nm relative to time‐matched controls (24 h set to 1). Data are means ± SEM from 7 independent experiments (4 replicates each). *p ≤ 0.05, **p ≤ 0.01, ***p ≤ 0.001 vs. 24 h control (one‐way ANOVA). (B) Growth of HGrC1 cells co‐expressing all four ERβ isoforms (4β) and corresponding controls was evaluated under the same conditions. Data are expressed as in (A). Means ± SEM from 7 independent experiments (4 replicates each). **p ≤ 0.01, ***p ≤ 0.001 vs. 24 h control (one‐way ANOVA). (C) Expression of EGR1, MYC, CTNNB1, BAX, and BCL2 was quantified by RT‐qPCR in ERβ isoform‐expressing cells or 4β cells and their respective controls, and normalized to GAPDH. Results are expressed as fold change vs. control (set to 1). The BAX/BCL2 ratio is shown. Data are means ± SEM from 4 to 5 independent experiments performed in triplicate. *p ≤ 0.05, **p ≤ 0.01, ***p ≤ 0.001 vs. control (one‐way ANOVA). A.U., arbitrary units.

Interestingly, in the 4β‐expressing line, both *EGR1* and *MYC* mRNA levels were significantly and markedly reduced (~0.7‐fold; *p* ≤ 0.01 and ~0.75‐fold relative to CTL; *p* ≤ 0.001, respectively, Figure 1C), consistent with the reduced growth observed in these cells (Figure 1B). CTNNB1 expression and the *BAX/BCL2* mRNA ratio remained unchanged. Since ERβ1 and ERβ4 alone did not significantly affect *EGR1* expression, one could propose that their heterodimerization with ERβ2 or ERβ5 inhibits ERβ2/ERβ5‐induced *EGR1* expression. Furthermore, despite the significant positive effects of ERβ2 and ERβ5 alone on *EGR1* and *MYC* expression (as well as on *CTNNB1* for β5), the 4β‐expressing line displayed down‐regulation of *EGR1* and *MYC*, with unchanged *CTNNB1* levels.

In conclusion, our results clearly demonstrate that ERβ2 and ERβ5, when expressed individually, promote cell proliferation notably through upregulation of proliferative genes that were downregulated by ERβ1 and ERβ4. However, when all four ERβ isoforms are co‐expressed, the inhibitory effects of ERβ1 and ERβ4 predominate, thereby contributing to the reduced cell growth in the 4β cell line. Our findings highlight the complex regulatory interplay among ERβ isoforms in GCs physiology and underscore the critical importance of ERβ isoform balance in determining biological outcomes.

### 3.2 Characterization of Transcriptional Activities of ERβ Isoforms in HGrC1 Cells

To elucidate the molecular mechanisms underlying the transcriptional activities of ERβ isoforms and their contribution to biological functions, particularly cell growth, we assessed both ligand‐dependent and ligand‐independent ERβ activities using luciferase reporter assays. Reporter gene expression was driven either by classical ERE‐ or AP‐1‐responsive promoters. In addition, to better mimic the physiological ERβ:ERα ratio observed in hGCs (~6:1) [44], we also examined the impact of ERβ/ERα co‐expression.

#### 3.2.1 ERβ Isoforms Inhibit E2‐Dependent ERα Transactivation at ERE Sites

First, we transiently transfected Flag‐ERβ1, Flag‐ERβ2, Flag‐ERβ4 or Flag‐ERβ5 in HGrC1 cells to validate their expression. As shown in Figure 2A, all isoforms were expressed at their expected molecular weights (59, 55, 54, and 53 kDa, respectively). Immunofluorescence analysis confirmed the nuclear localization of all ERβ isoforms, as demonstrated by co‐staining with DAPI (Figure 2B). Next, we evaluated their transactivation potential, in the absence or presence of 10 nm E2, using an ERE‐driven luciferase reporter assay. HGrC1 cells were transfected with Flag‐ERβ2, Flag‐ERβ4, or Flag‐ERβ5 alone or in combination with either Flag control vector (CTL), Flag‐ERβ1, or Flag‐ERα at varying receptor concentration ratios. These ratios were selected to reflect the relative ER expression balance observed in mature human GCs (ERα:ERβ ~1:6, and ERβ1:ERβ2/4/5 ~1:1 for each isoform) [44]. Importantly, transfection of an enhancer‐less vector (pGL2) did not produce detectable transcriptional activity in HGrC1 cells. As shown in Figure 2C, only Flag‐ERβ1 exhibited significant ligand‐dependent transactivation at ERE sites (~1.8‐fold compared to vehicle‐treated β1‐transfected cells; *p* ≤ 0.001). Flag‐ERβ1 also showed low ligand‐independent transactivation (~1.2‐fold relative to vehicle‐treated CTL; *p* ≤ 0.001), possibly reflecting basal transcriptional activity. In contrast, Flag‐ERβ2‐4‐5 exhibited no significant transactivation, either in the presence or absence of E2 (Figure 2C). Moreover, these isoforms did not affect ERβ1 transcriptional activity when co‐expressed at a 1:1 ratio (Figure 2D), consistent with our previous findings [44]. Figure 2E further shows that ERβ1 transcriptional activity remained unchanged under both treatment conditions when co‐expressed with ERβ2‐4‐5 (β_x_:β1 ratio of 3:1). These results indicate that ERβ2‐4‐5 do not interfere with ERβ1‐mediated transactivation at EREs.

**FIGURE 2:**
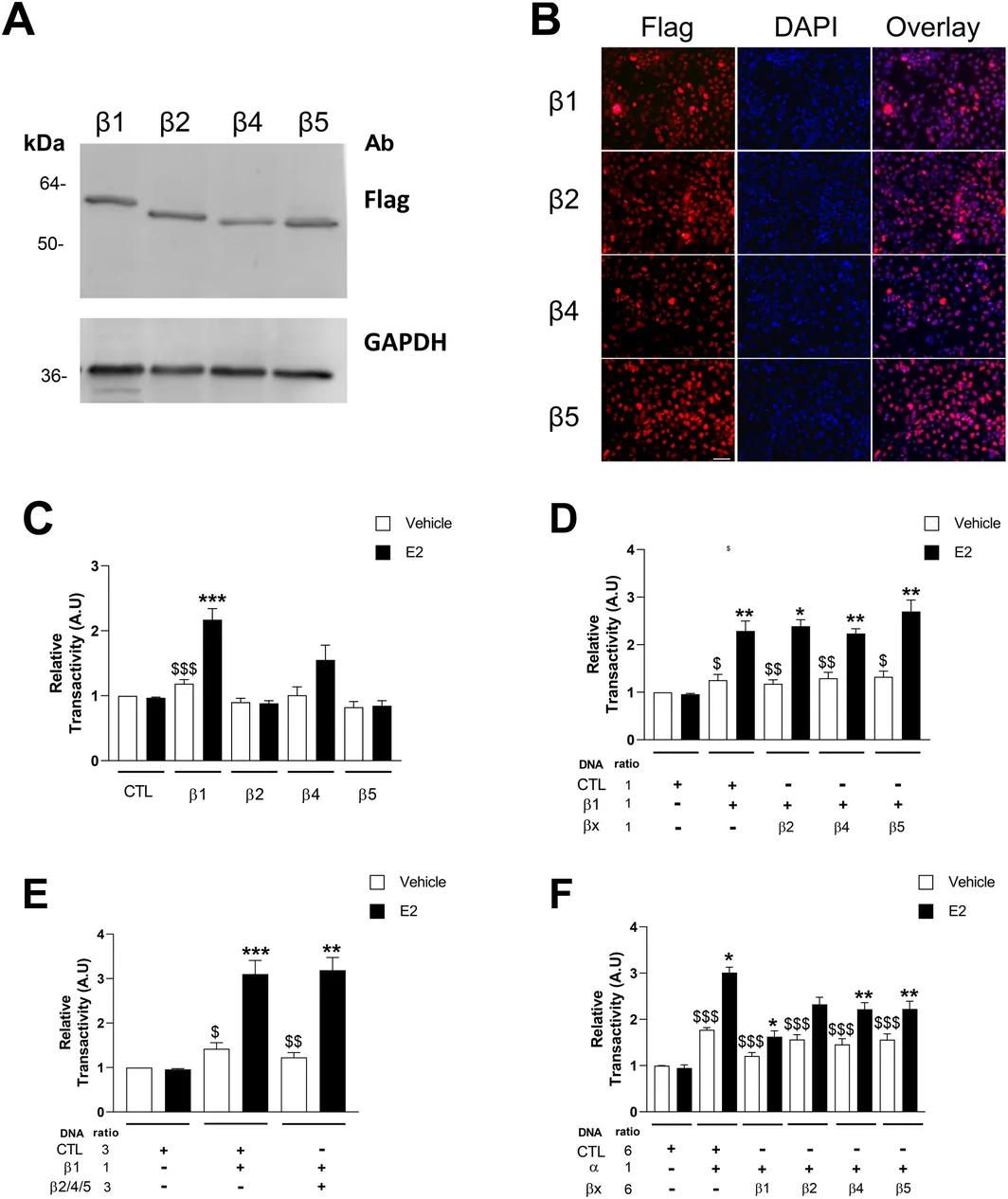
Each ERβ isoform inhibits E2‐induced ERα transactivation at ERE sites. (A, B) HGrC1 cells transiently expressing Flag‐ERβ1, ERβ2, ERβ4, or ERβ5 were analyzed by Western blot (anti‐Flag; GAPDH as loading control) and immunofluorescence. Representative cropped blots are shown in Figure S6. Immunofluorescence was performed 24 h post‐transfection using anti‐Flag antibody (red); nuclei were stained with DAPI (blue). Representative images (20×) are shown. (C–F) HGrC1 cells were transiently transfected with ERβ isoforms alone or in combination, or with ERα, at the indicated ratios, together with an ERE‐driven luciferase reporter (Renilla as internal control). Luciferase activity was measured 24 h after treatment with vehicle or 10 nM E2. Results are expressed as relative transactivation (CTL vehicle set to 1). The enhancer‐less vector (pGL2) showed no activity (not shown). (C) ERβ isoforms alone; (D) ERβ1 co‐expressed with individual ERβ isoforms (1:1); (E) ERβ1 co‐expressed with all ERβ isoforms (3:1); (F) ERα co‐expressed with ERβ isoforms (6:1). Data are means ± SEM from 7 to 11 independent experiments (six replicates each). *p ≤ 0.05, **p ≤ 0.01, ***p ≤ 0.001 vs. vehicle; ^$^ *p* ≤ 0.05, ^$$^ *p* ≤ 0.01, ^$$$^ *p* ≤ 0.001 vs. CTL vehicle (Mann–Whitney test). A.U., arbitrary units.

Then, we investigated whether ERβ isoforms modulate ERα‐mediated transcriptional activity at ERE sites, using a β_x_:α expression ratio of 6:1, reflecting the physiological context of mature GCs. As expected, Flag‐ERα significantly activated ERE‐driven luciferase expression, both in the presence (~3‐fold; *p* ≤ 0.05) and absence (~1.8‐fold; *p* ≤ 0.001) of E2, compared to vehicle‐treated ERα and CTL conditions, respectively (Figure 2F). Interestingly, co‐transfection of Flag‐ERα with individual ERβ isoforms did not affect ligand‐independent ERα activity (Figure 2F), but significantly reduced E2‐dependent ERα transactivation.

Collectively, our results demonstrate that in HGrC1 cells, only ERβ1 exhibits both ligand‐dependent and ligand‐independent transactivation at ERE sites. In addition, all ERβ isoforms exert inhibitory effects on E2‐dependent ERα activity while not affecting ERβ1‐mediated transactivation.

#### 3.2.2 ERβ2, ERβ4, or ERβ5 Prevents Ligand‐Independent ERβ1 Transrepression of AP‐1 Reporter Activity

We next investigated the role of ERβ isoforms in regulating AP‐1‐dependent gene expression, as ERα and ERβ have previously been reported to exhibit opposite, ligand‐dependent activities at AP‐1 sites in MCF‐7 and Ishikawa cells [4]. In those models, E2 enhances ERα‐mediated transactivation while promoting ERβ‐mediated transrepression of AP‐1 sites. To verify whether such a paradigm is relevant to all ERβ isoforms in HGrC1 cells, we transiently transfected HGrC1 cells with individual Flag‐ERβ isoforms or co‐transfected them with Flag‐ERβ1 or Flag‐ERα at varying concentration ratios. Transfection of the enhancer‐less vector (pGL4.4) resulted in no detectable reporter activity under the conditions tested. As shown in Figure 3A, Flag‐ERβ1 alone did not exhibit a ligand‐independent activity of AP‐1 reporter activity. However, in the presence of E2, it significantly reduced luciferase activity (~0.3‐fold decrease compared to vehicle‐treated β1‐transfected cells; *p* ≤ 0.001; Figure 3A), indicating ligand‐dependent transrepression. In contrast, the ligand‐insensitive isoforms Flag‐ERβ2‐4‐5 displayed significant ligand‐independent transactivation (~1.4‐, 1.2‐, and 1.2‐fold compared to vehicle‐treated CTL, respectively) (Figure 3A). As expected, E2 treatment did not alter the transcriptional activity of the ligand‐insensitive isoforms. Then, to investigate whether ERβ1 activity on AP‐1 reporter activity is influenced by the other isoforms, we co‐transfected Flag‐ERβ1 with individual Flag‐ERβ_x_ isoforms (*x* = 2, 4, or 5) at a 1:1 ratio (Figure 3B), or with all isoforms at a ratio of 3:1 ratio (Figure 3C). When co‐transfected alone (1:1 ratio with control vector), ERβ1 exhibited a weak but significant ligand‐independent transrepression (~0.13‐fold compared to vehicle‐treated CTL; *p* ≤ 0.001) (Figure 3B). This transrepression was completely abolished when ERβ1 was co‐expressed with ERβ2‐4‐5, indicating that these isoforms can counteract ERβ1‐mediated repression under basal conditions (Figure 3B). ERβ also mediated transrepression in the presence of E2, which was unaffected by co‐expression of any of the other ERβ isoforms (~0.2‐fold; *p* ≤ 0.01 reduction in luciferase activity compared to each respective vehicle‐treated co‐transfection condition) (Figure 3B). Strikingly, co‐expression of ERβ1 with all ERβ isoforms did not abolish the ligand‐independent transrepression activity of ERβ1 (Figure 3C), as observed with expression of a single ERβ isoform (Figure 3B). Instead, a modest increase in basal AP‐1 driven transcription was observed (~1.12‐fold increase compared to vehicle‐treated CTL; *p* ≤ 0.05) (Figure 3C). This effect may reflect the combined ligand‐independent transactivation capacity of ERβ2‐4‐5. In the presence of E2, co‐expression of all ERβ isoforms resulted in significant transrepression at AP‐1 sites, with a higher degree than that observed in the CTL:ERβ1 condition (~0.25‐fold vs. ~0.12‐fold decrease compared to respective vehicle‐treated condition) (Figure 3C). Together, these results indicate that ERβ2‐4‐5 can attenuate ERβ1‐mediated ligand‐independent transrepression at AP‐1 sites, likely through their intrinsic ligand‐independent transactivation activity.

**FIGURE 3:**
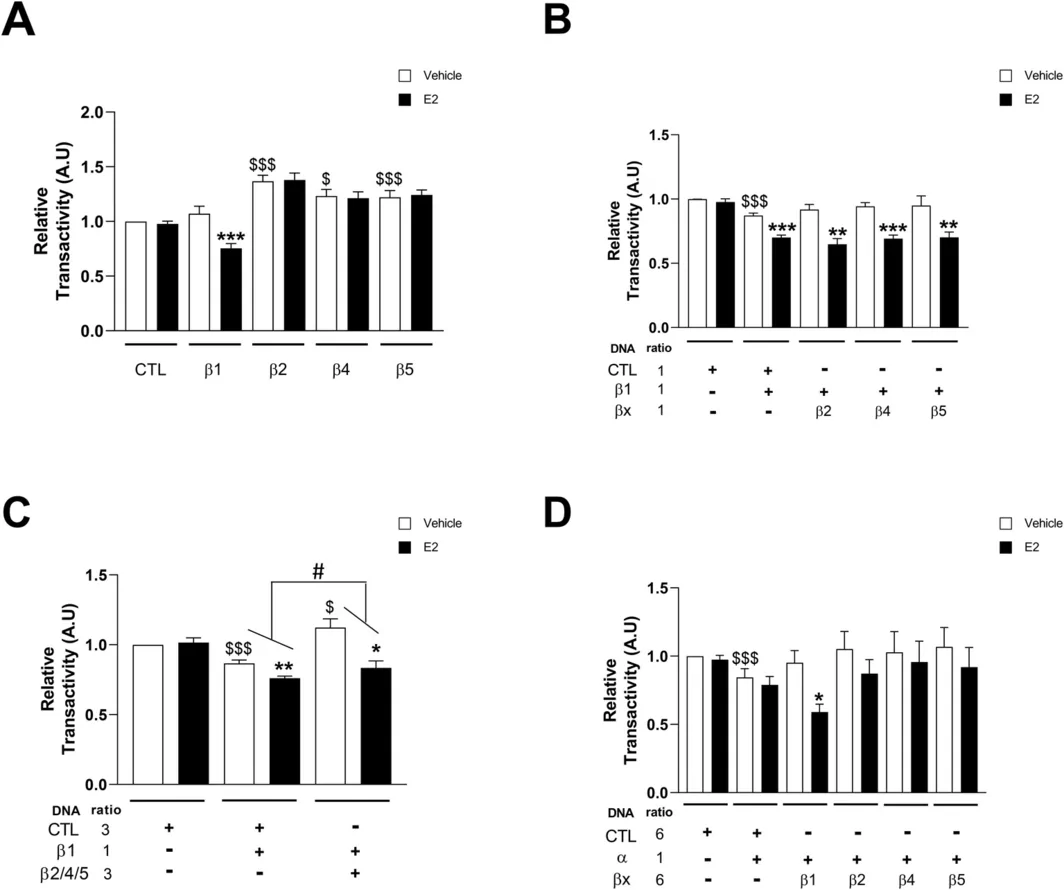
ERβ2, ERβ4 or ERβ5 prevents ligand‐independent ERβ1 transrepression at AP‐1 sites. HGrC1 cells were transiently transfected with Flag‐ERβ1, ERβ2, ERβ4, or ERβ5, alone or in combination, or with ERα, at the indicated ratios. Cells were co‐transfected with an AP‐1‐driven luciferase reporter (AP‐1‐Luc) or an enhancer‐less control vector (pGL4.44), together with a Renilla plasmid for normalization. Luciferase activity was measured 24 h after treatment with vehicle or 10 nM E2. Results are expressed relative to CTL vehicle (set to 1). The enhancer‐less vector showed no activity (not shown). (A) ERβ isoforms expressed individually; (B) ERβ1 co‐expressed with individual ERβ isoforms (1:1); (C) ERβ1 co‐expressed with all ERβ isoforms (3:1); (D) ERα co‐expressed with ERβ isoforms (6:1). Data are means ± SEM from 6 replicates in 6–10 independent experiments. *p ≤ 0.05, **p ≤ 0.01, ***p ≤ 0.001 vs. vehicle; ^$^ *p* ≤ 0.05, ^$$$^ *p* ≤ 0.001 vs. CTL vehicle; ^#^ *p* ≤ 0.05 vs. E2/vehicle fold‐change within groups (Mann–Whitney test). A.U., arbitrary units.

Finally, we assessed the transcriptional activity of Flag‐ERα on AP‐1 reporter activity in HGrC1 cells using a β:α expression ratio of ~6:1 as present in mature GCs. Surprisingly, ERα induced a modest but significant ligand‐independent transrepression (~0.94‐fold reduction compared to vehicle‐treated CTL; *p* ≤ 0.001) (Figure 3D), as observed for ERβ1 (Figure 3B). In contrast to previous reports in other cell types [4, 58], E2 treatment did not significantly alter ERα activity at AP‐1 sites in HGrC1 cells. In the presence of E2, transrepression was observed only when both ERβ1 and ERα were co‐expressed (~0.38‐fold decrease compared to CTL‐β1:α; *p* ≤ 0.001) (Figure 3D), likely reflecting the dominant repressive activity of ERβ1, as previously depicted in Figure 3B. Notably, the ligand‐independent transrepression mediated by ERα was abolished upon co‐expression of ERβ1, ERβ2, ERβ4, or ERβ5.

Overall, our results demonstrate that, unlike ERβ isoforms, ERα has a limited role in regulating AP‐1‐dependent transcriptional in HGrC1 cells. In contrast, ERβ1 acts as a key repressor, particularly in the presence of ligand, whereas ERβ2‐4‐5 promote ligand‐independent transcriptional activation that potentially attenuates ERβ1‐mediated repression. Although these findings are based on a reporter assay, they highlight isoform‐specific functional interactions that may contribute to AP‐1‐dependent gene regulation in a cell‐type‐specific manner.

### 3.3 Identification of Distinct Nuclear and Extra‐Nuclear Signaling Mechanisms Mediating ERβ Isoform Activities

Our above analyses indicate that ERβ1 is the predominant transcriptional regulator in HGrC1 cells while ERβ2‐4‐5 mainly modulate ERα activities at ERE sites and promote ligand‐independent transcriptional activity via AP‐1 sites. However, ERβ1‐specific target genes in GCs remain unknown, and the extra‐nuclear mechanisms underlying the effects of all ERβ isoforms on cell growth have yet to be characterized.

#### 3.3.1 Characterization of a Novel ERβ1 Transcriptional Signature in hGCs

While transcriptomic studies in rodent GCs have identified multiple ERβ target genes [59, 60, 61], ERβ1‐specific targets in humans remain largely unknown. Notably, whether co‐expression of ERβ2‐4‐5 influence the ERβ1‐driven transcriptome is an area that remains unexplored. Therefore, to characterize the transcriptional signature of ERβ1, we performed global transcriptomic profiling using RNA sequencing (RNA‐seq) on primary cultures of hGCs isolated from ovarian follicles of five women undergoing in vitro fertilization (L1–L5). *ESR2v1* encoding ERβ1 was selectively silenced using a lentiviral shRNA‐based knockdown approach, while control cells were transduced with a scrambled shRNA vector (S) (Figure 4A). As shown in Figure 4B, *ESR2v1* mRNA levels were significantly reduced in ERβ1 knockdown cells (B1), with ~60% decrease compared to scramble controls (S; *p* ≤ 0.01). Additionally, when compared to parental hGCs, depleting *ESR2v1* transcripts did not significantly affect the expression of other estrogen receptors confirming the specificity of the knockdown (Figure 4C). Principal component analysis (PCA) of global gene expression profiles revealed that samples clustered according to individual patients, indicative of inter‐individual variability (Figure 4D). Within this distribution, samples from L1 and L3 tended to segregate from those of L2, L4, and L5 along the principal components. This could not be attributed to differences in body mass index or basal hormone levels (Table S4). Stimulation‐related parameters were heterogeneous across patients, and no single parameter clearly accounted for the PCA separation. However, their potential contribution cannot be excluded given the small sample size. Further analysis identified 27 genes significantly upregulated in L1 and L3 compared to L2, L4, and L5 (FC > 2; *p* ≤ 0.01; Table S5). Among these, MAP3K3, GCH1, and SLC3A2 have been previously reported as E2‐responsive genes [62, 63, 64], suggesting inter‐individual differences in estrogen responsiveness.

**FIGURE 4:**
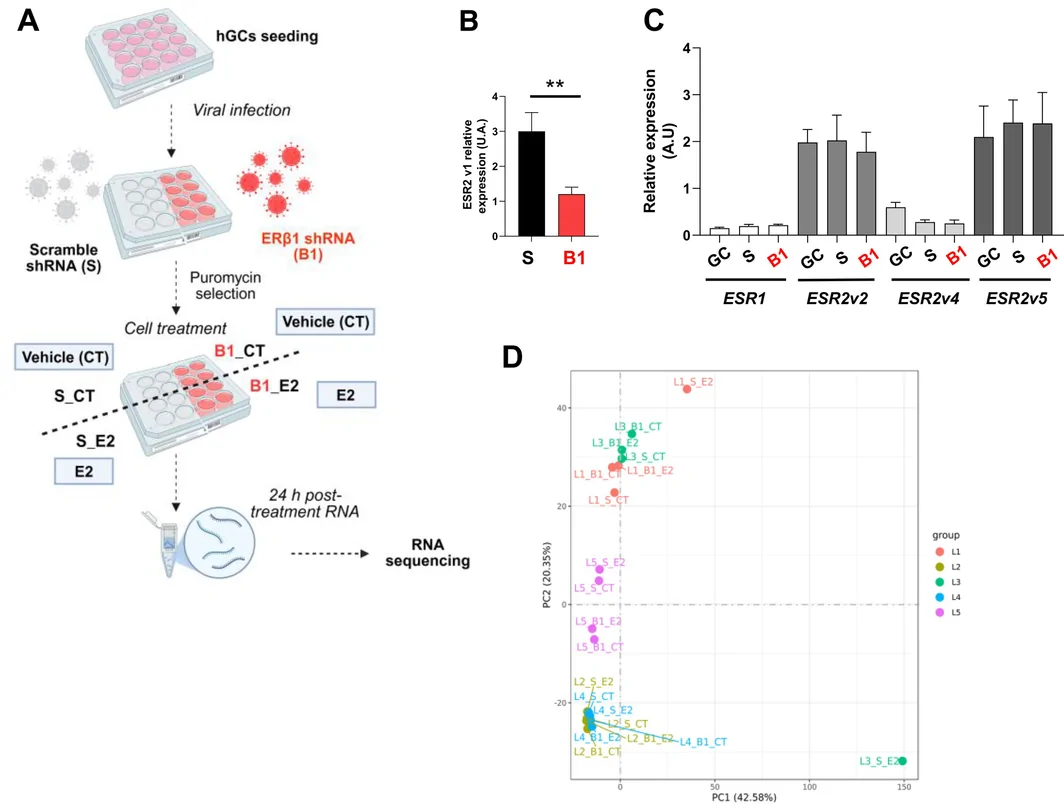
Principal component and RT‐qPCR analyses of ERβ1 knockdown and E2 treatment in primary human granulosa lutein cells. (A) Experimental design: Primary human granulosa lutein cells (hGCs) from five patients (L1‐L5) were transduced with shRNA targeting ERβ1 (B1) or scramble control (S), selected with puromycin, and treated with vehicle (CT) or 10 nM E2 for 24 h before RNA‐seq analysis (S_CT, S_E2, B1_CT, B1_E2). (B) Validation of ERβ1 knockdown by RT‐qPCR (ESR2v1) normalized to GAPDH. Data are means ± SEM from duplicates. **p ≤ 0.01 vs. S. (C) Expression of ESR1, ESR2v2, ESR2v4 and ESR2v5 in transduced hGCs compared with parental cells, normalized to GAPDH. Data are means ± SEM from all patient samples (4 conditions per patient). (D) Principal component analysis (PCA) of RNA‐seq data. Each dot represents one sample, colored by patient (L1–L5). PC1 and PC2 explain 42.58% and 20.35% of variance, respectively, and reveal clustering by patient and treatment condition consistency.

We next sought to identify E2‐responsive genes in hGCs by comparing gene expression profiles in ERβ1 expressing cells, treated with E2 (S_E2) or vehicle (S_CT). A volcano plot of differentially expressed genes (DEGs) between these two conditions is shown in Figure 5A (upper left panel). Using DESeq2 package with thresholds of FC > 2 and adjusted *p*‐value (*p*‐adj) ≤ 0.05, we identified 76 DEGs. Among these, 19 genes were upregulated after E2 treatment in all five patient samples while 57 genes were strongly upregulated (log_2_FC > 20) in samples L1 and L3 (Figure 5A); in agreement with a potential higher estrogen responsiveness. In those two samples, E2 markedly induced the expression of olfactory receptor genes (e.g., *OR1I1* and *OR4N2*), genes encoding motor or structural proteins (*MYT13*, *MYH7*, and *TUBB3P1*), as well as genes with diverse reported roles in endocrine signaling, transcriptional regulation and cellular growth‐related processes, including CSH1, CTCFL, and ENAM (Figure 5A). In all hCG samples, E2 upregulated *CYP3A4* and *SHANK2*, as previously reported [65, 66], and also increased the expression of less‐characterized targets, such as channel‐related genes *ANO2*, *GRIK4*, *CACNA1*, and *RBM20* (Figure 5A). Gene Ontology (GO) analysis of upregulated genes revealed significant enrichment in biological processes related to voltage‐ and ligand‐gated ion channel activity, transmembrane transporter activity and motor activity (Figure 5B), supporting a role for E2 in regulating ion flux and cellular dynamics.

**FIGURE 5:**
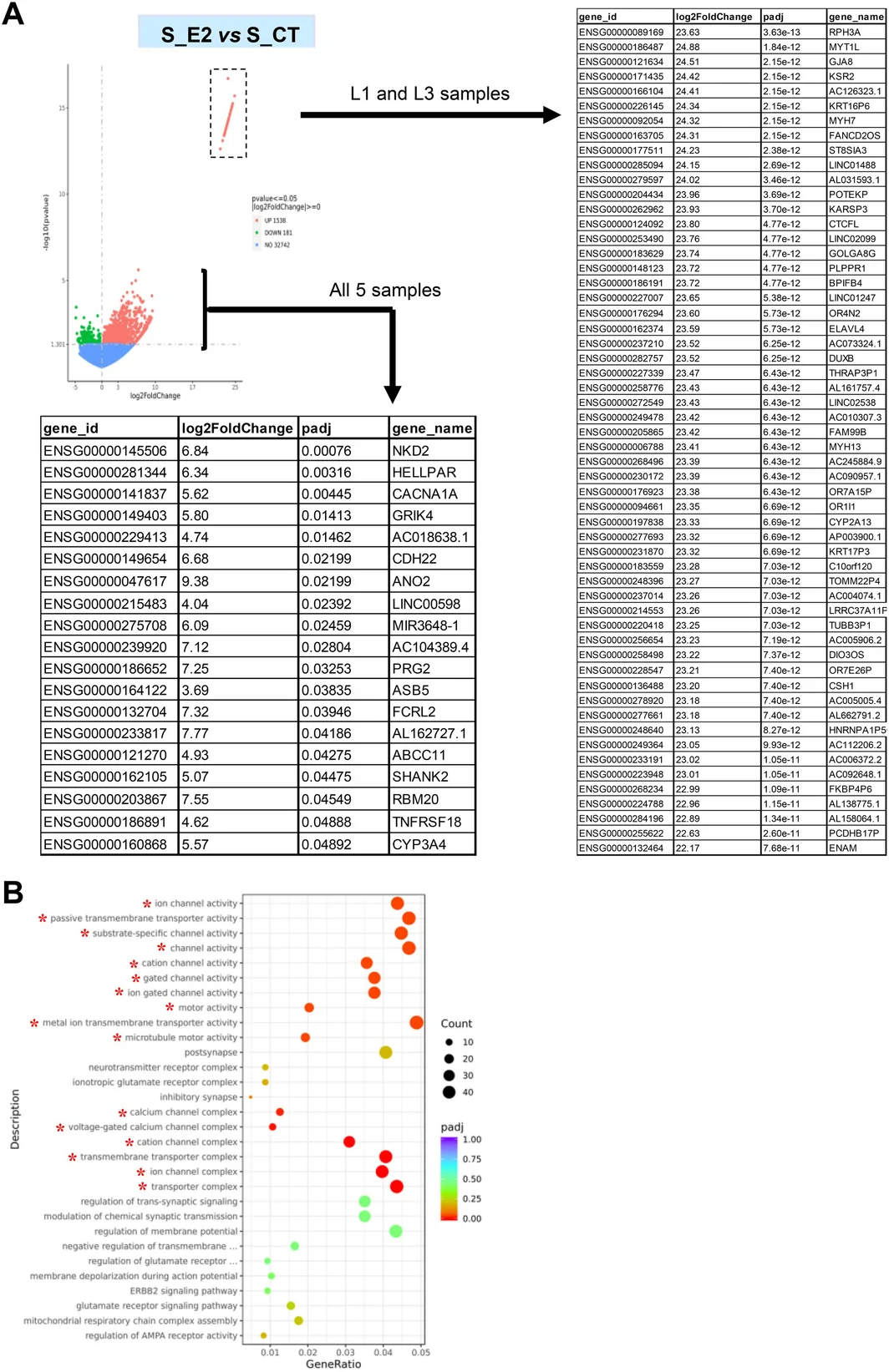
E2‐induced changes in the gene expression profile of hGCs. (A) Volcano plot showing differential gene expression between scramble‐transduced hGCs treated with vehicle (S_CT) or 10 nM E2 (S_E2). The *x*‐axis represents log_2_ fold change and the *y*‐axis the −log_10_ (*p*‐value). Upregulated genes (log_2_FC > 1, *p* ≤ 0.05) are shown in red, downregulated genes (log_2_FC < −1, *p* ≤ 0.05) in green, and non‐significant genes in blue. Patient‐specific responses were observed, with a cluster of strongly regulated genes in L1 and L3 (dashed box). Upper right panel: Genes upregulated only in L1 and L3 (log_2_FC > 2; *p*_adj_ ≤ 0.05). Bottom left panel: Genes upregulated across all patients (log_2_FC > 2; *p*_adj_ ≤ 0.05). (B) Gene Ontology (GO) enrichment analysis of differentially expressed genes. The ratio of DEGs per GO term is shown on the *x*‐axis, and GO terms are listed on the *y*‐axis. Dot size reflects gene number and color indicates significance. Biological processes with *p*_adj_ ≤ 0.05 are indicated by red stars.

We then investigated the effect of ERβ1 depletion (B1) on E2‐mediated transcriptional responses in hGCs. Comparisons of E2‐treated and CT conditions in ERβ1 knockdown cells (B1_E2 vs. B1_CT) did not identify significant DEGs at *p*_adj_ ≤ 0.05 (Figure S7A), indicating that ERβ1 is required for the full transcriptional response to E2 in this model. Similarly, no significant differences were observed between B1_CT and S_CT conditions (Figure S7B), suggesting that ERβ1 has limited ligand‐independent transcriptional activity under these conditions. To further assess the contribution of ERβ1 to E2 responsiveness, we compared gene expression between ERβ1 depleted (B1_E2) and scramble (S_E2) groups following E2 stimulation. As showed in the volcano plot (Figure 6A), the DEG profile revealed an overall inverse pattern compared to S_E2 vs. S_CT (Figure 5A). Indeed, E2‐induced genes in S_E2 were downregulated in B1_E2, indicating that E2 fails to activate gene transcription in the absence of ERβ1. Using DESeq2 with thresholds of FC < −2 and *p*_adj_ ≤ 0.05, we identified 26 genes downregulated in all samples (Figure 6B) and 50 genes in L1 and L3 samples (Figure S7C). Among the 26 common genes, 11 (highlighted in green) likely represent direct ERβ1 targets as they were previously identified as E2‐induced genes in scramble‐transduced cells (see Figure 5A bottom left panel). Notably, several of these genes encode ion channel‐related proteins, including *CACNA1A*, *GRIK4*, and *ANO2*, which are implicated in cellular growth‐related processes and cell division [67]. GO analysis (Figure 6C) of these downregulated genes revealed a significant reduced enrichment in ion channel activities, membrane potential regulation and motor activity, mirroring the upregulated pathways observed in S_E2 vs. S_CT (Figure 5B). KEGG pathway analysis further suggested the involvement of ERβ1 in pathways related to taste transduction and glutamatergic synaptic signaling, as well as adrenergic signaling and cAMP pathways (*p*_adj_ ≤ 0.05) (Figure 6D). Due to the low availability of ERβ1‐deleted hGCs, we rather validated ERβ1‐dependent transcriptional regulation using selected target genes in a stable HGrC1 cell line expressing ERβ1. RT‐qPCR analysis confirmed a significant increase in mRNA levels of *CACNA1A* (~1.14‐fold; *p* ≤ 0.05), *GRIK4* (~2.02‐fold; *p* ≤ 0.01), and *ANO2* (~1.48‐fold; *p* ≤ 0.001) in ERβ1‐expressing cells compared to control cells (CTL) (Figure 6E).

**FIGURE 6:**
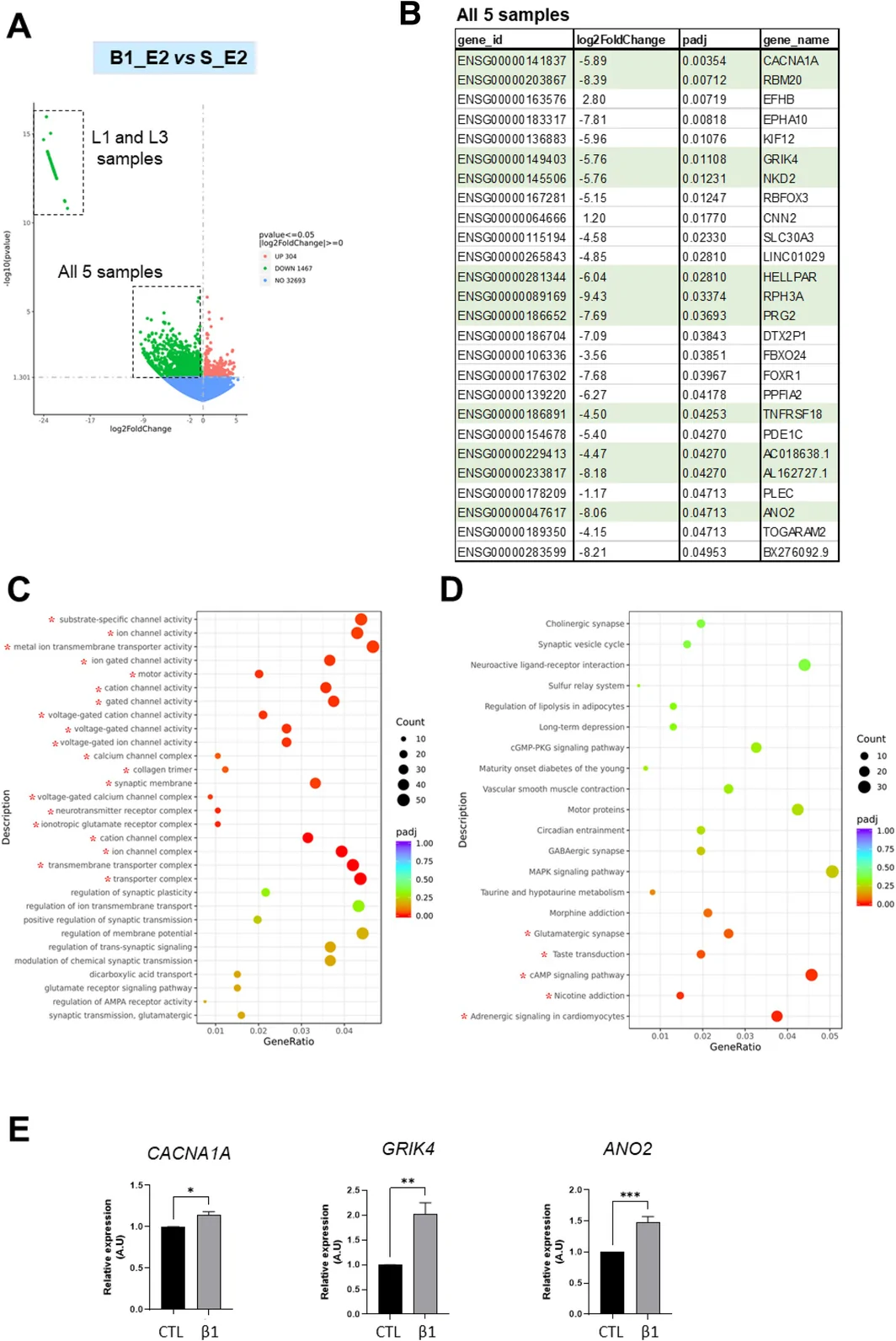
Differential gene expression analysis between E2‐treated scramble and ERβ1 shRNA‐transduced hGCs. (A) Volcano plot showing differential gene expression between E2‐treated scramble (S_E2) and ERβ1 knockdown (B1_E2) hGCs. The *x*‐axis indicates log_2_ fold change and the *y*‐axis −log_10_(*p*‐value). Upregulated genes (log_2_FC > 1, *p* ≤ 0.05) are shown in red, downregulated genes (log_2_FC < −1, *p* ≤ 0.05) in green, and non‐significant genes in blue. A patient‐specific cluster of differentially expressed genes is highlighted for L1 and L3 (dashed box). (B) Genes significantly downregulated in B1_E2 compared to S_E2 across all five patients (log_2_FC < −2; *p*_adj_ ≤ 0.05). Genes in green were also regulated in the opposite direction in the S_E2 vs. S_CT comparison (Figure 5A). (C) Gene Ontology (GO) enrichment analysis of DEGs. The *x*‐axis shows the proportion of DEGs per GO term, and the *y*‐axis lists GO terms. Dot size reflects gene number and color indicates enrichment significance. (D) KEGG pathway enrichment analysis of DEGs. The *x*‐axis indicates the proportion of DEGs per pathway, the *y*‐axis lists pathways. Dot size reflects gene number and color indicates statistical significance. Biological processes and pathways with *p*_adj_ ≤ 0.05 are indicated by red stars. (E) RT‐qPCR validation of CACNA1A, GRIK4 and ANO2 expression in ERβ1‐expressing HGrC1 cells versus controls. Data are means ± SEM from 4 to 7 independent experiments. *p ≤ 0.05, **p ≤ 0.01, ***p ≤ 0.001 vs. CTL (Mann–Whitney test).

Collectively, our data identify ERβ1 as a key mediator of E2‐induced transcriptional activity in hGCs. We define a set of novel ERβ1‐dependent, E2‐responsive genes, many of which are involved in ion transport and channel activity, processes that are essential for GC functions and oocyte support [68, 69].

#### 3.3.2 ERβ1, ERβ2, and ERβ4, but not ERβ5, Activate Distinct Extra‐Nuclear Signaling Pathways in HGrC1 Cells

To further investigate the potential extra‐nuclear mechanisms of ERβ isoforms, we analyzed activation of key extra‐nuclear signaling pathways involved in cell growth, such as ERK1/2, Akt, and CaMKII [70, 71] in HGrC1 cells stably expressing either a single or all four ERβ isoforms under basal conditions. Protein extracts were analyzed by Western blot to assess the phosphorylation status of these kinases. Both total and phosphorylated forms of each protein were detected (Figure S1), and signal intensities were normalized to total protein levels (for ERK1/2 and Akt) or to GAPDH (due to the lack of reliable antibodies against total CaMKII). Each ERβ‐expressing clone was analyzed relative to its matched control cell line, generated under the same selection conditions and results were expressed as the ratio of the signal in ERβ‐expressing cells to that of their corresponding control (each control set to 1). This normalization strategy accounts for differences in basal protein levels between control cell lines (such as β1 and β5 clones), which may arise from distinct selection conditions.

As shown in Figure 7, expression of ERβ1 or ERβ4 resulted in a significant increase in ERK1/2 phosphorylation (~1.8‐fold or ~2.6‐fold compared with CTL, respectively; *p* ≤ 0.01), whereas ERβ2 and ERβ5 had no significant effect. Akt phosphorylation was significantly enhanced in HGrC1 cells expressing ERβ2 and ERβ4 (~1.6‐fold and 2.1‐fold increases compared to CTL, respectively; *p* ≤ 0.05), while ERβ1 and, again, ERβ5 showed no significant impact. Notably, ERβ1 and ERβ2 expression led to a marked increase in CaMKII phosphorylation (~5.7‐fold and 3.6‐fold relative to CTL, respectively; *p* ≤ 0.01).

**FIGURE 7:**
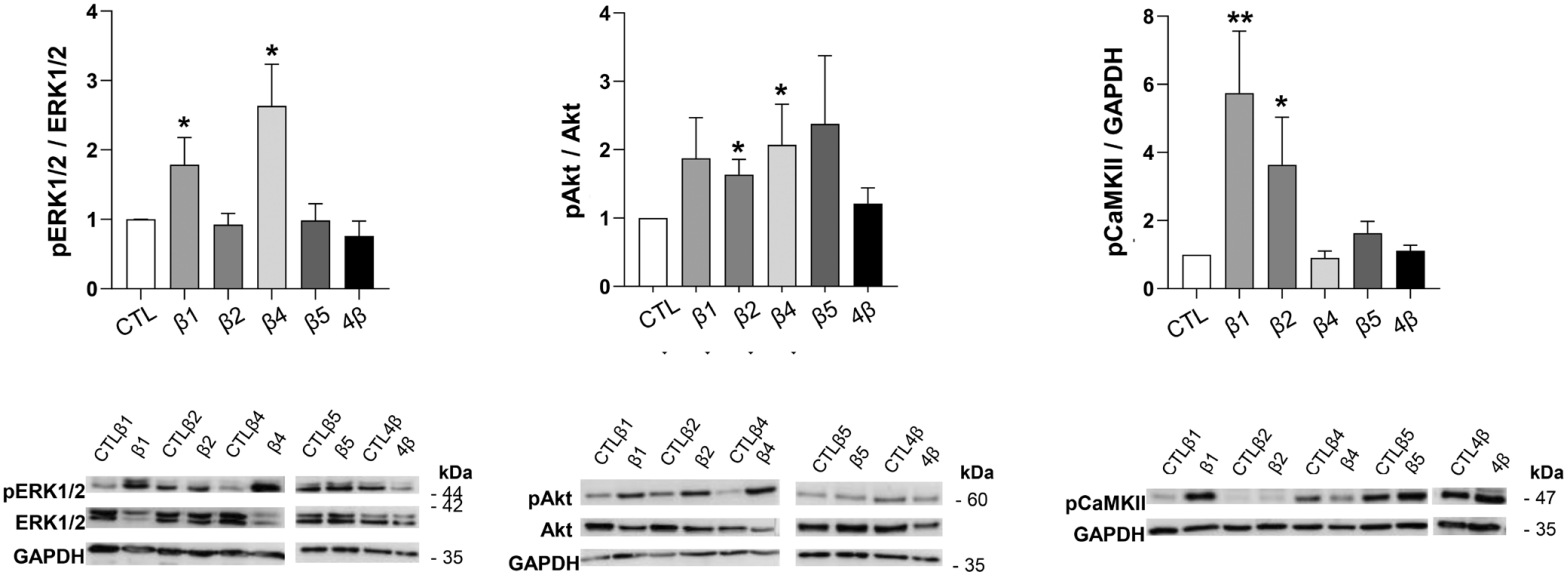
Differential activation of extra‐nuclear signaling pathways by ERβ isoforms in HGrC1 cells. Phosphorylation of ERK1/2 (left), Akt (middle), and CaMKII (right) was analyzed by Western blot in HGrC1 cells stably expressing individual ERβ isoforms (β1, β2, β4, β5) or all isoforms (4β), together with their respective controls under basal conditions. Phosphorylated proteins were normalized to total ERK1/2 and Akt levels or to GAPDH for CaMKII, and values were expressed relative to matched controls (set to 1). Data are means ± SEM from 4 to 8 independent experiments. *p ≤ 0.05, **p ≤ 0.01 vs. matched control (Mann–Whitney test). Representative cropped blots are shown below each graph. Corresponding full blots are presented in Figure S1.

Interestingly, those signaling pathways were not activated in HGrC1 cells co‐expressing all ERβ isoforms, suggesting that heterodimerization between ERβ isoforms may restrain their engagement in extra‐nuclear signaling and instead favor genomic signaling.

Collectively, our results indicate that ERβ1 and ERβ4 promote ERK1/2 activation, possibly contributing to their anti‐proliferative effects. In contrast, the pro‐proliferative isoform ERβ2 preferentially activates both Akt and CaMKII. ERβ5 did not significantly activate any of the signaling pathways analyzed in HGrC1 cells under basal conditions, in contrast to previous observations in a lung‐derived cell model [40]. This discrepancy may reflect differences in cellular context, including the availability of specific co‐factors, highlighting the cell‐type specificity of ERβ signaling [40]. Overall, these findings underscore the importance of ERβ isoform balance in shaping downstream signaling outcomes.

#### 3.3.3 ERβ Isoforms Have Distinct Interactomes in HGrC1 Cells

To further investigate the signaling pathways underlying the distinct biological roles of ERβ isoforms in HGrC1 cells, we carried out a complementary approach to identify isoform‐specific interacting partners. ERβ2‐4‐5 differ from the full‐length ERβ1 by the deletion of the last 61 C‐terminal amino acids containing the AF‐2 domain, which are replaced by unique extensions of 26, 12, and 3 residues, respectively [5]. We hypothesized that these structural variations alter receptor conformation, thereby promoting isoform‐specific protein interactions and providing a mechanistic basis for their distinct biological effects.

To identify the ligand‐dependent interactome of ERβ1 (the only isoform retaining ligand‐binding capacity), cells expressing tagged‐ERβ1 were treated with either vehicle (V) or E2 for 24 h prior to immunoprecipitation (IP) followed by LC–MS/MS analysis. In parallel, each Flag‐tagged ERβ isoform was expressed individually in HGrC1 cells to compare isoform‐specific interactome in HGrC1 cells. A Flag‐empty vector was used as a negative control to exclude non‐specific interactions. All samples were processed and analyzed in parallel. The bait protein (Flag‐ERβ) was reliably identified, with comparable label‐free quantification coverage (~46.4%) and a similar number of unique peptides (~56) across all conditions. In total, we identified 138 high‐confidence interactors across the different ERβ isoforms. These interactors are summarized in Venn diagrams showing (i) ERβ1 interactors lost upon E2 binding (Figure 8A) and (ii) shared and isoform‐specific interactors (Figure 8B).

**FIGURE 8:**
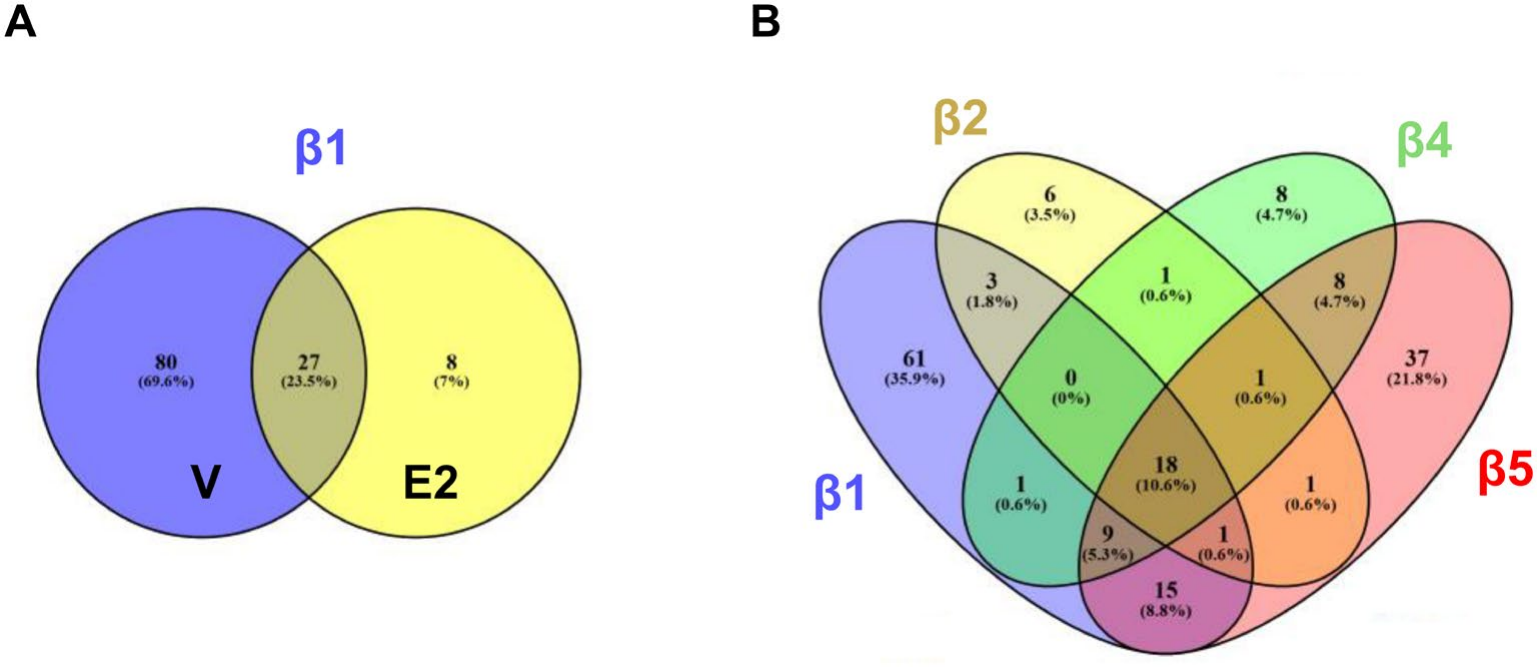
Identification of ERβ isoform‐specific interactomes in HGrC1 cells. (A) Venn diagram showing shared and condition‐specific ERβ1‐interacting proteins identified under vehicle (V) and E2 treatment conditions. (B) Venn diagram showing shared and isoform‐specific interacting proteins identified for ERβ1, ERβ2, ERβ4, and ERβ5.

Notably, our mass spectrometry approach validated several previously reported ERβ interactions identified using biochemical methods such as IP and GST pull‐down assays, including NCOR1 [72], VASP [73], FXR [74], and HSP90AB1 [75]. The molecular chaperone HSPA90A is known to interact with both ERα and ERβ in the absence of ligand, with dissociation occurring upon ligand binding. Consistently, ERβ1 interacted with HSP90A under vehicle (Figure 9A; Table S6) but not E2 (Figure 9B) conditions. In the presence of E2, ERβ1 specifically interacted with distinct nuclear proteins (HMGN1, PTMS, and ZMYM3), proteins associated with membrane trafficking (RAB3GAP1, VPS18) and microtubules (PRC1), as well as a co‐receptor that modulates growth factor signaling (GPC1). Analysis of ERβ1 protein interaction network under vehicle conditions was generated using STRING database and revealed interconnected clusters, with HSP90A emerging as a central interaction hub (Figure 9A; Table S7). Among the 11 clusters identified, proteins were associated with chaperone complexes, RNA modification in both the nucleus and cytosol, cytosolic ribosomal subunits, COPI‐independent Golgi‐to‐ER retrograde trafficking and the tricarboxylic acid cycle. As shown in Table 1A, we identified a shared core set of 17 interactors common to all four ERβ isoforms. Among them, 10 represent novel potential partners (highlighted in green) that are not reported in the BioGRID or ENCODE/Harmonizome 3.0 interaction databases. These include proteins involved in transcriptional regulation and RNA splicing (RPAP3, AAR2), cell growth and intracellular organization (DRG2, ARHGAP21, NEDD4, TBCE) and cellular energy metabolism (AK3). Notably, HSPA90A was not identified as a binding partner of ERβ2, ERβ4, and ERβ5, thereby excluding them from a large number of protein interactions observed for ERβ1 (Figure 9A). The inability of these truncated isoforms to interact with HSP90A is consistent with the requirement for the full‐length LBD for HSP90 interaction [76]. Interestingly, ERβ isoforms differ in protein stability, with ERβ5 showing increased susceptibility to proteasomal degradation [35]. The lack of HSP90 association may therefore contribute to reduced chaperone‐mediated stabilization and differential turnover of the truncated ERβ isoforms.

**FIGURE 9:**
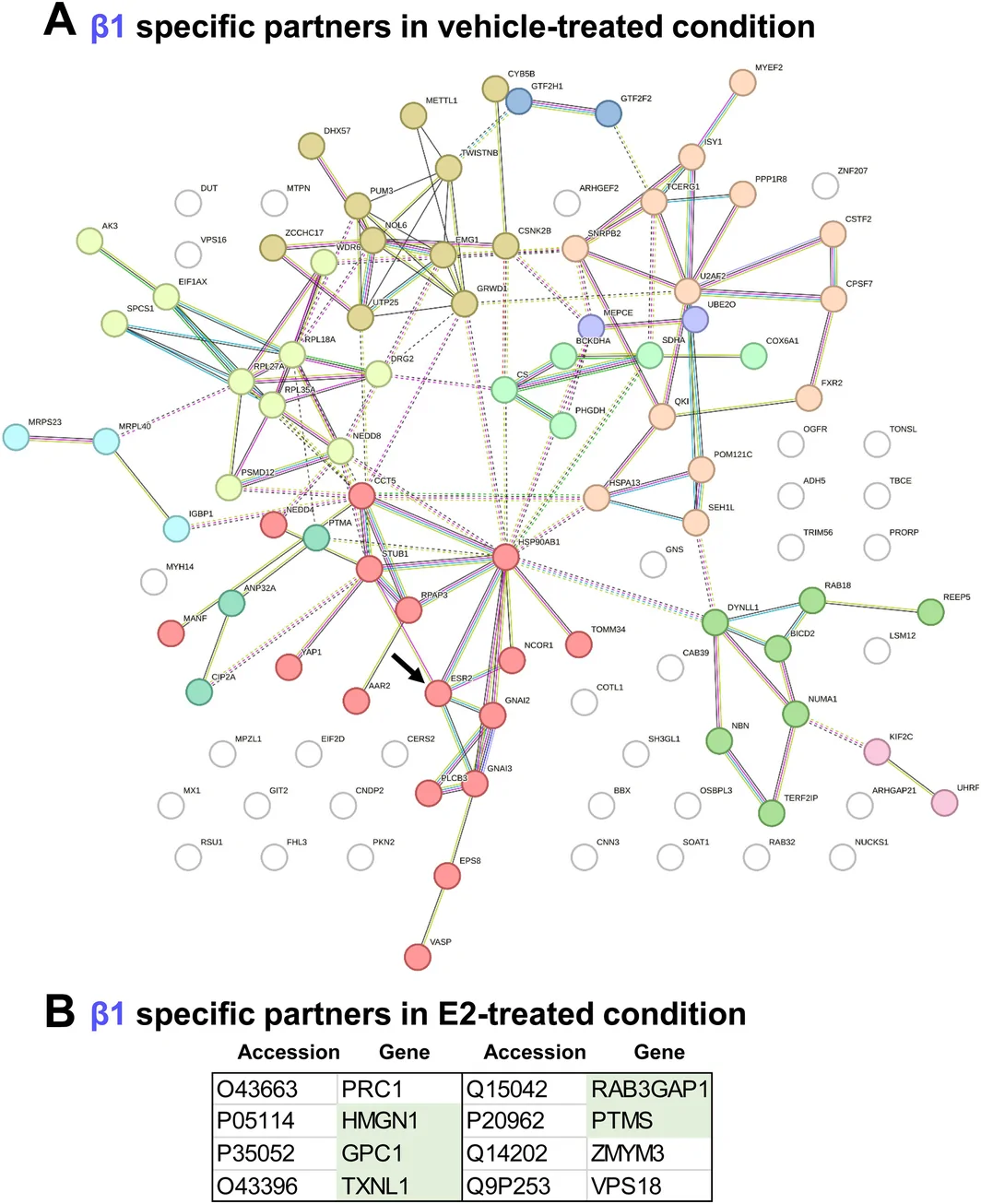
STRING‐based clustering of ERβ1‐specific interactors in the absence or presence of E2. (A) Protein–protein interaction network generated using the STRING database from ERβ1‐specific interactors identified under vehicle conditions. Nodes represent proteins and colors indicate cluster assignment. Edges correspond to known or predicted functional associations (confidence score ≥ 0.7). (B) ERβ1‐specific interacting proteins identified following E2 treatment. Proteins highlighted in green represent potential novel interactors not reported in the BioGRID database. Accession numbers and gene names are indicated, and the arrow identifies ESR2 (ERβ).

**TABLE 1:** Common and specific interactors of ERβ isoforms identified by mass spectrometry.

Accession	Gene
**(A) Common interactants**	
P20591	MX1
Q5T5U3	ARHGAP21
Q9H6T3	RPAP3
Q96G23	CERS2
P32780	GTF2H1
P58546	MTPN
P46934	NEDD4
Q9C0C9	UBE2O
Q9Y312	AAR2
P04899	GNAI2
P55039	DRG2
Q9UIJ7	AK3
Q15813	TBCE
P15586	GNS
P33240	CSTF2
Q9NP72	RAB18
Q9NYB0	TERF2IP
**(B) Specific interactants**	
*β1*	
P08238	HSP90AB1
P06454	PTMA
Q14980	NUMA1
P48643	CCT5
Q96T88	UHRF1
Q15785	TOMM34
P50552	VASP
O14776	TCERG1
P31040	SDHA
P33316	DUT
O00232	PSMD12
Q92974	ARHGEF2
O75390	CS
P63167	DYNLL1
Q02543	RPL18A
P35610	SOAT1
P13984	GTF2F2
P51116	FXR2
Q15843	NEDD8
P18077	RPL35A
O43175	PHGDH
Q15417	CNN3
O75376	NCOR1
Q96PU8	QKI
P67870	CSNK2B
Q96EE3	SEH1L
P46776	RPL27A
Q14161	GIT2
Q96KP4	CNDP2
Q9GZS3	SKIC8
Q9Y3D9	MRPS23
O15091	PRORP
Q13637	RAB32
P08579	SNRPB2
P47813	EIF1AX
Q9Y376	CAB39
Q8N684	CPSF7
O95297	MPZL1
Q9NQ50	MRPL40
Q16513	PKN2
Q92979	EMG1
Q7L2J0	MEPCE
Q00765	REEP5
O60934	NBN
Q15404	RSU1
O43670	ZNF207
Q12972	PPP1R8
P12694	BCKDHA
P08754	GNAI3
Q9BQ67	GRWD1
Q8TCG1	CIP2A
Q9H1E3	NUCKS1
Q3MHD2	LSM12
Q8WY36	BBX
Q6P158	DHX57
Q9UNE7	STUB1
Q15397	PUM3
Q99961	SH3GL1
Q68CQ4	UTP25
P48723	HSPA13
A8CG34	POM121C
Q3B726	POLR1F
Q9H269	VPS16
P55145	MANF
P46937	YAP1
P33240	CSTF2
Q9P2K5	MYEF2
Q14019	COTL1
Q7Z406	MYH14
Q9BRZ2	TRIM56
Q9H4L5	OSBPL3
Q96HA7	TONSL
Q9UBP6	METTL1
P78318	IGBP1
Q9ULR0	ISY1
Q9NZT2	OGFR
P41214	EIF2D
Q9H6R4	NOL6
Q13643	FHL3
Q01970	PLCB3
*β2*	
Q96EY7	PTCD3
O95232	LUC7L3
P54727	RAD23B
Q96SI9	STRBP
Q6P4E1	GOLM2
Q8WUD4	CCDC12
*β4*	
Q99733	NAP1L4
P21980	TGM2
Q9NNW5	WDR6
Q6PJF5	RHBDF2
Q9GZT8	NIF3L1
O95786	RIGI
O43665	RGS10
P09960	LTA4H
*β5*	
Q99439	CNN2
Q9NSV4	DIAPH3
Q8N1F7	NUP93
P30084	ECHS1
Q3KQU3	MAP7D1
Q9NTI5	PDS5B
Q7Z7H5	TMED4
Q96JM3	CHAMP1
P21589	NT5E
P23921	RRM1
O94760	DDAH1
Q9Y316	MEMO1
O14981	BTAF1
P50897	PPT1
Q9NVS9	PNPO
O43768	ENSA
Q13098	GPS1
Q14320	FAM50A
Q9UBT2	UBA2
Q5H9R7	PPP6R3
Q13011	ECH1
Q9Y570	PPME1
P53367	ARFIP1
Q09472	EP300
Q9NP61	ARFGAP3
P46527	CDKN1B
Q96C19	EFHD2
Q9UM00	TMCO1
Q01085	TIAL1
Q9NY27	PPP4R2
O14818	PSMA7
Q13618	CUL3
Q96TA1	NIBAN2
Q15042	RAB3GAP1
P51003	PAPOLA
Q9BRT2	UQCC2
A0MZ66	SHTN1

*Note:* (A) Interacting proteins shared among all four ERβ isoforms. (B) Isoform‐specific interacting proteins identified for ERβ1, ERβ2, ERβ4, and ERβ5. Proteins highlighted in green represent potential novel interactors not reported in the BioGRID database. Accession numbers and gene names are indicated.

In addition, distinct subsets of isoform‐specific interactors were identified (Table 1B): 61 for ERβ1, 6 for ERβ2, 8 for ERβ4 and 37 for ERβ5. As showed in Figure 10, ERβ2 specifically interacted with a limited number of proteins involved in gene expression and mRNA processing (PCTD, LUC7L3, CCDC12), protein degradation (RAD23B) and vesicular trafficking (GOLM1). Similarly, ERβ4 displayed eight potential specific interactors, including a modulator of G protein‐coupled signaling pathways (RGS10), nuclear proteins with potential transcriptional regulator functions (NAP1L4 and NIF3L1), and the multifunctional protein scaffold TGM2, which is involved in cell adhesion, extracellular remodeling and cell survival [77]. Finally, ERβ5 exhibited 37 potential specific interactors, including transcriptional regulators (EP300, BTAF1), proteins associated with intracellular membrane‐bound organelles, such as the Golgi (RAB3GAP1, ARFIP1, ARFGAP3) and mitochondria (UQCC2, ECHS1, ECH1), as well as proteins involved in actin and tubulin polymerization (CNN2, DIAPH3, MAP7D1).

**FIGURE 10:**
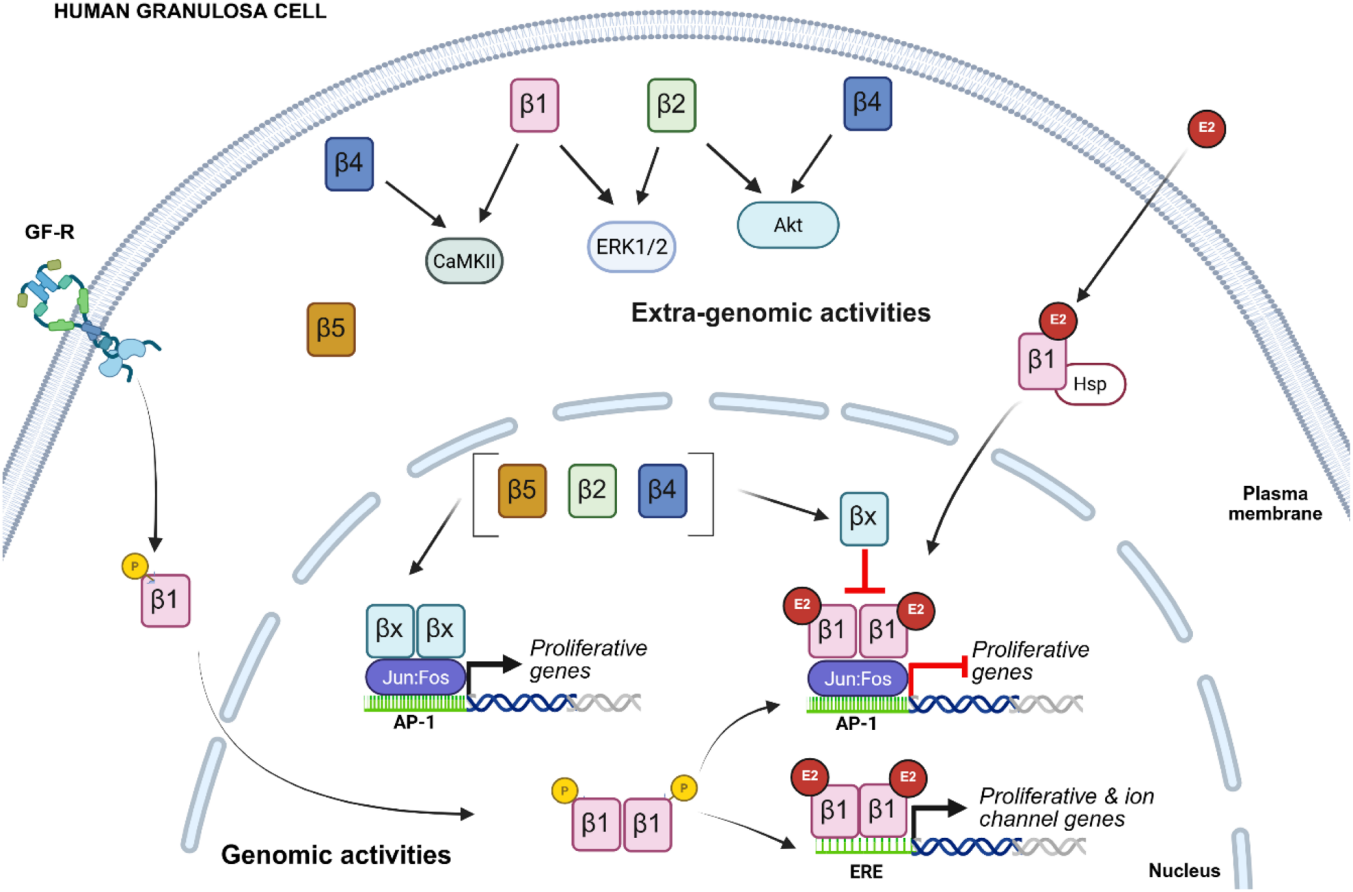
Proposed model of nuclear and extra‐nuclear ERβ isoform signaling in hGCs. ERβ isoforms regulate granulosa cell function through integrated nuclear and extra‐nuclear signaling pathways. Following E2 binding, ERβ1 dissociates from heat shock proteins (HSPs) and promotes ERE‐dependent transcription or represses AP‐1‐dependent transcription through interactions with AP‐1 complexes. In contrast, ERβ2 (β2), ERβ4 (β4), and ERβ5 (β5) are ligand‐insensitive and lack ERE‐dependent transcriptional activity. These isoforms can promote ligand‐independent AP‐1‐mediated transcription and attenuate ERβ1‐dependent repression, potentially following phosphorylation by growth factor receptor (GF‐R)‐associated kinase pathways. In parallel, ERβ isoforms differentially activate extra‐nuclear signaling pathways including CaMKII, ERK1/2, and Akt. Through isoform‐specific interactions and crosstalk, these signaling mechanisms ultimately determine GC growth outcomes. Created in BioRender. https://BioRender.com. 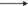 Activation; 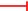 repression; 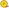 phosphorylation.

Altogether, our findings provide the first comprehensive mapping of isoform‐specific ERβ interactomes in GCs, revealing both shared and distinct protein partners. Beyond their canonical nuclear functions, ERβ isoforms may also modulate mitochondrial activity, intracellular trafficking and cytoskeletal dynamics, thereby increasing the molecular diversity of estrogen signaling in GCs and potentially contributing to their role in supporting oocyte maturation.

## 4 Discussion

ERβ signaling in ovarian GCs is highly complex, reflecting the coexistence of genomic and non‐genomic mechanisms and the expression of multiple human‐specific ERβ isoforms with divergent C‐terminal structures. These isoforms differ in ligand binding, dimerization capacity, transcriptional activity, and protein–protein interactions, suggesting that their relative expression and crosstalk critically shape E2 responses. In this study, we combined functional, transcriptomic, and proteomic approaches to dissect the isoform‐specific roles of ERβ in human GCs.

### 4.1 Functional Interplay Among ERβ Isoforms Controls Granulosa Cell Growth and Signaling

Our results demonstrate that ERβ isoforms exert distinct functions in HGrC1 cells: ERβ1 and ERβ4 reduce cell growth, whereas ERβ2 and ERβ5 promote it, consistent with observations in cancer models, where ERβ1 displays tumor‐suppressive properties while ERβ2 and ERβ5 are associated with enhanced proliferation and invasion [26, 29, 41]. Notably, when all ERβ isoforms were co‐expressed, the anti‐proliferative features predominated, indicating that ERβ1 and ERβ4 act epistatically over the others, and that signaling crosstalk between ERβ isoforms modulates cellular outcomes. This functional interplay was also evident at the level of extra‐nuclear signaling. Expression of individual isoforms selectively activated ERK1/2, Akt, or CaMKII, whereas their co‐expression prevented activation of these pathways in our cell model (Figure 10). Altogether, these findings suggest that the relative balance of ERβ isoforms constrains signaling outputs, potentially through heterodimerization, altered receptor conformation, or competition for shared signaling intermediates, thereby favoring genomic mechanisms and/or scaffold‐mediated functions. Supporting this hypothesis, analysis of ERβ isoform interactomes revealed that each isoform interacts with proteins involved in membrane trafficking, mitochondrial metabolism, and cytoskeletal organization, features likely contributing to the regulation of GC growth under these experimental conditions. It should be noted that our cell model lacks endogenous ERα, suggesting that additional regulatory layers may influence ERβ signaling in a physiological context. Nonetheless, our data indicate that the relative balance of ERβ isoforms, as established in our model, plays a central role in regulating cell signaling and growth. A similar mechanism may operate in vivo, although further studies at physiological expression levels will be required to further substantiate these findings.

### 4.2 AP‐1‐Dependent and Ligand‐Independent Transcription as a Major Mode of ERβ Isoform Genomic Action

In contrast to ERβ1, the three isoforms ERβ2‐4‐5 were unable to activate ERE‐dependent transcription in HGrC1 cells, likely due to truncation of their LBD, which compromises stable homodimerization and recruitment of transcriptional co‐regulators. Nevertheless, all ERβ isoforms repress ERα‐mediated transcription at EREs, as previously reported in other cell models [38], possibly through competition for DNA binding or sequestration of co‐activators such as TIP60 [78]. Whether ERβ2, ERβ4, and ERβ5 form heterodimers with ERα remains to be established and will be essential to clarify the reduced transcriptional competence of such complexes.

Importantly, using AP‐1 reporter assays, we demonstrate for the first time in GCs that, contrary to ERβ1, which represses AP‐1‐mediated transcription, ERβ2‐4‐5 increase AP‐1‐reporter activity (Figure 10). Identifying the molecular determinants underlying these differential effects may help elucidate the distinct mechanisms of action of ERβ isoforms in GCs. Moreover, ERβ2‐4‐5 attenuated ERβ1‐mediated AP‐1 repression, further revealing functional antagonism among ERβ isoforms. This contrasts with findings in neuronal cells [36], highlighting the cell‐type specificity of ERβ/AP‐1 signaling. Mechanistically, ERβ isoforms may enhance AP‐1 activity through AF‐1‐dependent interactions with p160 co‐activators within AP‐1 complexes [79, 80], sequestration of AP‐1 associated repressors or TIP60 recruitment facilitated by ligand‐independent N‐terminal phosphorylation [11]. Collectively, our data based on AP‐1 reporter assays, reinforce the concept that AP‐1 dependent, ligand‐independent mechanisms can serve as an important mode of ERβ action in GCs. This mode of action may be particularly relevant under physiological conditions characterized by low or fluctuating E2 levels, such as early folliculogenesis and reproductive aging. ERβ2‐4‐5 may in particular contribute to early follicle growth, when follicles have not yet started producing E2. Moreover, our findings prompt a reassessment of current views on E2 signaling in the postmenopausal period, a stage commonly assumed to involve minimal estrogenic activity due to the absence of ovarian E2 production. Our results suggest that ERβ2‐4‐5, through ligand‐independent activity, may sustain AP‐1‐mediated transcriptional programs under low‐E2 conditions, thereby potentially contributing to endocrine regulation beyond classic E2 signaling.

### 4.3 E2‐Dependent Transcriptional Regulation of hGC Is Mediated Through ERβ1

Our transcriptomic analyses identify ERβ1 as a central component of a GC‐specific transcriptional program that cannot be compensated by ERα. This may be explained by the fact that, as demonstrated, ERβ2‐4‐5 are able to antagonize ERα‐mediated ERE‐dependent transcriptional activity.

We further demonstrate that E2, acting through ERβ1, upregulates genes enriched in pathways controlling plasma membrane electrical potential (mainly through ion transport and ion channel activity), cytoskeletal organization, motor activity and ion channel transport, including *CACNA1A*, *ANO2*, and *GRIK4*. These ion channels have been previously reported to be expressed in GCs [68, 81, 82] and are critical for Ca^2+^, Cl^−^, and Na^+^ influxes, which regulate steroidogenesis, oocyte maturation and ovulation [81, 82]. Calcium release from intracellular stores has also been shown to be mediated by ERβ1 in neuronal cells [83]. To support follicular functions, such as cell growth and steroidogenesis, ERβ1 activation may increase the expression of these channels, while their targeting to the plasma membrane may be coordinated by cytoskeletal components. Accordingly, ERβ1 interacts with ARHGAP21 and TBCE, which regulate actin and tubulin cytoskeletons, respectively, as well as with proteins involved in COPI‐independent Golgi‐to‐ER retrograde trafficking. This suggests that ERβ1 may coordinate protein trafficking in the absence of E2, subsequently modulating protein synthesis upon ligand binding. However, these hypotheses require further investigation.

Pathways analysis of E2‐activated ERβ1‐mediated gene expression highlighted cAMP and ionotropic/metabotropic signaling pathways. These findings point to a broader role of ERβ1 in integrating extra‐nuclear and nuclear signals to regulate GC metabolic and steroidogenic functions that support oocyte growth.

The distinct cellular and hormonal contexts of HGrC1 cells and primary hGCs may contribute to differences in ERβ1 responses between the two models. Thus, the ERβ1‐associated reduction in growth observed in HGrC1 cells should not be directly equated with the E2‐induced expression of growth‐related genes observed in primary hGCs. Previous studies in other cellular systems have similarly highlighted the context‐dependent nature of ERβ1 activity, and notably the influence of ERα [84]. These complementary models therefore allow ERβ signaling to be examined both through controlled isoform‐specific functional assays in HGrC1 cells and through E2‐dependent transcriptional responses in differentiated human GCs.

### 4.4 Isoform‐Specific Interactomes Reveal Divergent Signaling Modalities

Proteomic analyses further revealed that each ERβ isoform associates with a distinct interactome, comprising both nuclear transcriptional co‐factors and proteins involved in extra‐nuclear functions. The ability of ERβ isoforms to simultaneously interact with multiple functionally diverse partners suggests that they may act as scaffold‐like proteins, coordinating isoform‐specific signaling and biological activities. These findings expand the current view of ERβ functions by revealing potential interactions with proteins involved in cytoskeletal remodeling, membrane dynamics and mitochondria metabolism.

Specifically, ERβ1 preferentially associated with proteins regulating actin cytoskeletal dynamics (ARHGEF2) and vesicular trafficking (Rab32), consistent with a role in protein transport and spatial signaling organization. ERβ1 was also linked to mitochondrial metabolism and redox regulation through interactions with SHDA and CS, in line with the reported stimulatory effects of E2 on several mitochondrial enzyme expression and ATP production [85], as well as the upregulation of nuclear DNA‐encoded COX subunit I expression by ERβ1 [86]. ERβ2 interactors are enriched in nuclear proteins involved in RNA processing (PTCD3, LUC7L3, RAD23B, and STRBP) and Golgi‐associated proteins (GOLM2), pointing to potential roles in post‐transcriptional regulation and protein trafficking. ERβ4 selectively interacted with regulators of GPCR (RGS10) and integrin signaling (TGM2), consistent with its ability to activate ERK1/2 and Akt pathways, which may indirectly influence genomic responses. Indeed, MAP‐kinase activity has been shown to contribute to ERα and ERβ‐mediated non‐genomic action at AP‐1 sites [19]. Although ERβ5 did not activate ERK1/2, Akt or CaMKII signaling pathways in GCs, it exhibited a broader interactome than ERβ2 and ERβ4. ERβ5 may interact with proteins involved in RNA maturation (EP300, FAM50A), vesicular trafficking (RAB3GAP1), actin and tubulin organization (CNN2, MAP7D1), mitochondrial energy metabolism (ECHS1, ECH1, UQCC2), phosphatase regulation (PP6R3, PP4R2, PPME1), calcium homeostasis (TMCO1) and nitric oxide production (DDAH1), suggesting multifaceted nuclear and extra‐nuclear functions of ERβ5 in GCs.

Although these interactomes were derived from a single overexpression experiment, they provide a valuable framework highlighting clear isoform‐specific differences, which can guide further validation at physiological expression levels. Together, these findings support a model in which ERβ isoforms act not only as transcription factors but also as scaffold‐like integrators of signaling pathways, thereby orchestrating distinct cellular responses in GCs, essential to support oocyte maturation.

## 5 Conclusions

Overall, our study highlights the potential significance of ERβ isoform balance in determining estrogen signaling outcomes and provides new insights into ERβ signaling in GCs. The frequent co‐expression of multiple ERβ isoforms in estrogen‐responsive tissues underscores the importance of dissecting isoform‐specific signaling mechanisms and the isoform crosstalk. A more precise characterization of the temporal and spatial expression of ERβ isoforms in GCs is now warranted; however, this will require the development of reliable isoform‐specific antibodies, which remain unavailable. Ultimately, a deeper understanding of ERβ isoform interplay will be essential for better elucidating estrogen action in both endocrine physiology and disease.

## Author Contributions

**Clémentine Marie:** data curation, formal analysis, validation, investigation, visualization, methodology. **Inza‐Noor Baskaran:** formal analysis, investigation, visualization. **Raphaël Corre:** methodology, investigation, formal analysis. **Anne Mayeur:** resources. **Michaël Grynberg:** resources. **Guillaume Chevreux:** methodology. **David L'Hôte:** methodology, formal analysis and funding acquisition. **Joëlle Cohen‐Tannoudji:** funding acquisition, writing review and editing. **Stéphanie Chauvin:** conceptualization, resources, data curation, formal analysis, supervision, validation, investigation, visualization, writing‐original draft, project administration, writing‐review and editing.

## Funding

This work was funded by “Institut National de la Santé & de la Recherche Médicale” (Inserm), “Centre National de la Recherche Scientifique” (CNRS), Université Paris Cité and by fellowships from Ecole Doctorale Bio‐SPC (C. Marie and I‐N Baskaran).

## Ethics Statement

Ethical approval was granted by the institutional review board (IRB Blefco) under registration number “2021‐1.” The date of the first subject enrollment is June 2021. All women were fully counseled. Written informed consent was obtained from all subjects for participation to this study and for publication of this paper. The study was conducted in accordance with the Declaration of Helsinki for Medical Research Involving Human Subjects (2013 revision).

## Consent

All patients consent for publication. All authors have approved the manuscript and agree with its submission.

## Conflicts of Interest

The authors declare no conflicts of interest.

## Supporting information


**Figure S1:** Representative Western blots for phosphorylated ERK1/2, Akt, and CaMKII, with corresponding controls in ERβ‐expressing HGrC1 cells. Proteins extracted from each β‐expressing HGrC1 line (ESR2 variants v1 (β1), v2 (β2), v4 (β4), v5 (β5) or all four isoforms (4β)), along with their respective control (CTL), were analyzed by Western blot. Blots were probed to detect: (A) phosphorylated and total ERK1/2 (P‐ERK and ERK, 42–44 kDa), (B) phosphorylated and total Akt (P‐Akt and Akt, 60 kDa), and (C) phosphorylated CaMKII (P‐CaMKII, 47 kDa) and GAPDH (37 kDa). The images are representative of at least 4 independent experiments. Blots marked with a red asterisk (*) indicate membranes previously probed with other antibodies; residual signals are visible but do not represent non‐specific bands.
**Figure S2:** Expression of Flag‐tagged ERβ isoforms in HGrC1 cells before immunoprecipitation. HGrC1 were transiently transfected with either Flag‐control, Flag‐ERβ1, Flag‐ERβ2, Flag‐ERβ4, or Flag‐ERβ5 for 48 h. Protein extracts were prepared as described in Section 2. An aliquot (40 μg) of each protein extract was used in Western blot analysis to detect Flag‐tagged proteins and GAPDH. ERβ1, ERβ2, ERβ4, and ERβ5 were detected at the expected molecular weights of 59, 55, 54, and 53 kDa, respectively.
**Figure S3:** Generation and validation of HGrC1 clonal cell Lines expressing a single ERβ isoform. (A) Protocol of establishment of HGrC1 clonal cell lines stably expressing individual ERβ isoforms (ESR2 variants v1 (β1), v2 (β2), v4 (β4), or v5 (β5)). Each isoform was associated with a specific fluorescent protein (EGFP, mCherry, TagBFP, and Citrine for β1, β2, β4, and β5, respectively) and a corresponding antibiotic resistant marker (puromycin, hygromycin B, neomycin, and zeocin, respectively). Control cell lines expressed only the respective fluorescent proteins alongside with the matching selection marker. Following electroporation, cells were selected using the corresponding antibiotic for 4–6 weeks. Monoclonal lines were then generated by limiting dilution, and positives clones were identified based on fluorescence. After clone expansion, transgene expression, as well as endogenous ESR2 isoform expression, were confirmed by RT‐qPCR as described in the Methods. Created in BioRender. https://BioRender.com/30ku5os. (B) Total mRNA was extracted from β1#4‐, β2#1‐, β4#4‐, and β5#1‐expressing clones, along with their corresponding control cell lines (CTL). Expression levels of transgenic ESR2 were quantified by RT‐qPCR and normalized to ACTB. Data are shown as means ± SEM from duplicates across 3 independent experiments.
**Figure S4:** Growth rate analysis and ESR2 variant expression in clonal cell lines. (A) Growth rate comparison between parental HGrC1 cells and control clones. The growth rates of parental (Par.) HGrC1 cells and clonal lines expressing only the fluorescent reporter proteins (CTLβ1#1, CTLβ2#2, CTLβ4#1, and CTLβ5#2) were assessed over 96 h. Formazan production, directly proportional to the number of viable cells, was measured by recording changes in absorbance at 575 nm. Fold changes in growth were calculated as the ratio of the ${\mathrm{OD}}_{575\;\mathrm{nm}}$ values at 48 h, 72 h, and 96 h relative to 24 h, with 24 h‐value set to 1 as a control. Of note, the number of cells was similar (${\mathrm{DO}}_{575}∼0.6$) for each cell line at 24 h. No significant differences in growth were observed. This indicates that the stable line engineering procedure did not affect HGrC1 cell growth. Data are representative of 4 independent experiments performed in quadruplicate. Data are presented as means ± SEM. *p ≤ 0.05, **p ≤ 0.01, ***p ≤ 0.001 vs. 24 h control, by ordinary one‐way ANOVA. A.U., Arbitrary Units. (B) Total RNA was extracted from the four single β‐expressing clones (β1#4, β2#1, β4#1, and β5#4), the 4β#7‐expressing clone and their respective control cell line (CTL). Expression levels of transgenic ESR2 variants (in β‐expressing clones) were quantified by semi‐quantitative RT‐PCR. PCR aliquots (5 μL) were collected at 20, 25, and 30 cycles. We got complete saturation after 35 cycles. PCR products were separated on a 5% agarose gel, stained with ethidium bromide and visualized under UV light. Expected amplicon sizes were 246 bp (ERβ1), 141 bp (ERβ2), 91 bp (ERβ4), and 71 bp (ERβ5). Signal quantification during the exponential phase (30 cycles) showed that all isoforms are expressed in the 4 β clone with a slightly higher expression level for β5 isoform. (C) Growth rates of two distinct 4β‐expressing clones (4β#7 and 4β#22) were assessed by MTT assay at 24, 48, 72, and 96 h post‐seeding. Fold change in growth for each 4β clone was calculated relative to the corresponding control line at each time point, with the 24 h value set arbitrary to 1 as a control. No significant difference was observed between 4β#7 and 4β#22 clones as both exhibited reduced growth 48 h post‐seeding that was maintained up to 96 h. Each experiment was repeated 5–7 times with quadruplicate measurements. Data represent means ± SEM. * *p* < 0.05, **p ≤ 0.01 vs. 24 h control, by the one‐way ANOVA with Dunn's post hoc test. A.U., Arbitrary Units.
**Figure S5:** Effect of transient expression of ERβ isoforms on HGrC1 cell proliferation. HGrC1 cells were transiently and efficiently (~70%) transfected by nucleofection with either Flag‐Cherry (CTL), Flag‐ERβ1 (β1), Flag‐ERβ2 (β2), Flag‐ERβ4 (β4), or Flag‐ERβ5 (β5). Cell proliferation was assessed by BrdU incorporation in Flag‐positive cells, detected by immunofluorescence. DAPI stains the nucleus. Magnification, ×20. Data represent means ± SEM from 4 replicates performed 4–8 times. **p ≤ 0.01, ***p ≤ 0.001 vs. CTL, by the one‐way ANOVA with Dunn's post hoc test.
**Figure S6:** Analysis of Flag‐ERβ isoform expression by western blotting. Western blot analysis was performed on HGrC1 cells transiently expressing Flag‐mCherry control, Flag‐ERβ1 (β1), Flag‐β2 (β2), Flag‐β4 (β4), or Flag‐β5 (β5). Protein extracts were probed for Flag (upper panel) and GAPDH (lower panel). All ERβ isoforms were expressed at their expected molecular weights (59, 55, 54, or 53 kDa, respectively), as well as the flagged mCherry fusion protein (32 kDa).
**Figure S7:** Analysis of differential gene expression in ERβ1‐depleted hGCs, treated or not with E2. (A) Volcano plot showing differentially expressed genes (DEGs) in hGCs transduced with ERβ1 shRNA (B1), treated with either vehicle (CT) or E2. (B) Volcano plot showing DEGs between hGCs transduced with scramble (S) or ERβ1 (B1) shRNA, both treated with vehicle (CT). In both panels, the *x*‐axis represents the log_2_ fold change ($\log_{2}\mathrm{FC}$) in gene expression between groups, indicating up‐ or downregulation. The *y*‐axis shows the $-\log_{10}\left(p\;\text{value}\right)$, reflecting the statistical significance of differential expression. Genes significantly upregulated ($\log_{2}\mathrm{FC}>1$, p<0.05) are shown in red; significantly downregulated genes ($\log_{2}\mathrm{FC}<-1$, p<0.05) are shown in green; and non‐significantly varying genes are displayed in blue. (C) List of DEGs between E2‐treated hGCs transduced with scramble (S) or ERβ1 (B1) shRNA in L1 and L3 samples (B1_E2 vs. S_E2). Genes highlighted in green are those found in common between the B1_E2 vs. S_E2 and S_E2 vs. S_CT comparisons (see Figure 5D).
**Table S1:** Primer sequences for ESR2 variants integration in the MXS vector.
**Table S2:** The shRNA target sequences and primer sequences.
**Table S3:** Primer sequences for qPCR.
**Table S4:** Clinical characteristics of the women selected for the study.
**Table S5:** Upregulated genes in patient samples L1 and L3, compared to patient samples L2, L4 and L5 based on RNA‐seq analysis. (Cells derived for each patient and transduced with scramble shRNA (S) were treated with either vehicle (control, CT) or E2, and gene expression levels were measured as mapped read counts. Gray columns represent read counts in hGCs from patients L1 and L3, while blue columns show read counts from patients L2, L4, and L5. The yellow column displays the average expression ratio between the two patient groups (L1 and L3 vs. L2, L4, and L5) after CT or E2 treatment, and the white column reports the corresponding *p*‐values from unpaired *t*‐tests. Only genes that were significantly upregulated (p<0.05) in both CT‐ and E2‐treated cells from patients L1 and L3, compared to the other patient group, are included.)
**Table S6:** MCL‐based protein clustering of ERβ1‐specific partners in the vehicle‐treated condition.
**Table S7:** Functional annotation of MCL clusters of ERβ1‐specific partners in the vehicle‐treated condition.

## Data Availability

Resource availability: Primary materials generated during this study are available upon reasonable request through the lead contact. Data and code availability: Mass spectrometry proteomics data have been deposited at ProteomeXchange Consortium via the PRIDE partner repository as dataset identifier PXD073667 and are publicly available as of the date of publication. RNA‐seq data has been deposited at ArrayExpress (EMBL' European Bioinformatics Institute) under the accession number E‐MTAB‐16651 and are publicly available as of the date of publication.
