# Supplementary material for: Estrogen Receptor Beta Isoforms Interplay Integrates Nuclear and Extra‐Nuclear Signaling to Modulate Human Granulosa Cell Growth
Source: FASEB J. 2026 Oct 3;40(19):e72328. doi: 10.1096/fj.202602705RR (PMC13633505; doi:10.1096/fj.202602705RR)
Supplement: Supplementary file 1 [file FSB2-40-e72328-s001.zip › FSB2_72328 Supporting information captions.pdf]

Supplementary Figure S1. Representative Western blots for phosphorylated ERK1/2, Akt and CaMKII, with corresponding controls in ERβ-expressing HGrC1 cells. Proteins extracted from each β-expressing HGrC1 line (ESR2 variants v1 (β1), v2 (β2), v4 (β4), v5 (β5) or all four isoforms (4β)), along with their respective control (CTL), were analysed by Western blot. Blots were probed to detect: (a) phosphorylated and total ERK1/2 (P-ERK and ERK, 42-44 kDa), (b) phosphorylated and total Akt (P-Akt and Akt, 60 kDa), and (c) phosphorylated CaMKII (P-CaMKII, 47 kDa) and GAPDH (37 kDa). The images are representative of at least 4 independent experiments. Blots marked with a red asterisk (*) indicate membranes previously probed with other antibodies; residual signals are visible but do not represent non-specific bands.

Supplementary Figure S2. Expression of Flag-tagged ERβ isoforms in HGrC1 cells before immunoprecipitation. HGrC1 were transiently transfected with either Flag-control, Flag-ERβ1, Flag-ERβ2, Flag-ERβ4 or Flag-ERβ5 for 48 h. Protein extracts were prepared as described in the Methods section. An aliquot (40 µg) of each protein extract was used in Western blot analysis to detect Flag-tagged proteins and GAPDH. ERβ1, ERβ2, ERβ4 and ERβ5 were detected at the expected molecular weights of 59, 55, 54 and 53 kDa respectively.

Supplementary Figure S3. Generation and validation of HGrC1 clonal cell Lines expressing a single ERβ isoform. (A) Protocol of establishment of HGrC1 clonal cell lines stably expressing individual ERβ isoforms (ESR2 variants v1 (β1), v2 (β2), v4 (β4) or v5 (β5)). Each isoform was associated with a specific fluorescent protein (EGFP, mCherry, TagBFP and Citrine for β1, β2, β4 and β5 respectively) and a corresponding antibiotic resistant marker (puromycin, hygromycin B, neomycin and zeocin, respectively). Control cell lines expressed only the respective fluorescent proteins alongside with the matching selection marker. Following electroporation, cells were selected using the corresponding antibiotic for 4-6 weeks. Monoclonal lines were then generated by limiting dilution, and positives clones were identified based on fluorescence. After clone expansion, transgene expression, as well as endogenous ESR2 isoform expression, were confirmed by RT-qPCR as described in the 4 Methods. Created in BioRender. (B) Total mRNA was extracted from β1#4-, β2#1-, β4#4- and β5#1-expressing clones, along with their corresponding control cell lines (CTL). Expression levels of transgenic ESR2 were quantified by RT-qPCR and normalized to ACTB. Data are shown as means ± SEM from duplicates across 3 independent experiments.

Supplementary Figure S4. Growth rate analysis and ESR2 variant expression in clonal cell lines. (A) Growth rate comparison between parental HGrC1 cells and control clones. The growth rates of parental (Par.) HGrC1 cells and clonal lines expressing only the fluorescent reporter proteins (CTLβ1#1, CTLβ2#2, CTLβ4#1 and CTLβ5#2) were assessed over 96 h. Formazan production, directly proportional to the number of viable cells, was measured by recording changes in absorbance at 575 nm. Fold changes in growth were calculated as the ratio of the OD575nm values at 48 h, 72h and 96 h relative to 24 h, with 24 h-value set to 1 as a control. Of note, the number of cells was similar (DO575 ~ 0.6) for each cell line at 24 h. No significant differences in growth were observed. This indicates that the stable line

engineering procedure did not affect HGrC1 cell growth. Data are representative of 4 independent experiments performed in quadruplicate. Data are presented as means ± SEM. * $P \leq 0.05$, ** $P \leq 0.01$, *** $P \leq 0.001$ vs. 24 h control, by ordinary one-way ANOVA. A.U., Arbitrary 6 Units. (B) Total RNA was extracted from the four single β-expressing clones (β1#4, β2#1, β4#1 and β5#4), the 4β#7-expressing clone and their respective control cell line (CTL). Expression levels of transgenic ESR2 variants (in β-expressing clones) were quantified by semi-quantitative RT-PCR. PCR aliquots (5 µL) were collected at 20, 25 and 30 cycles. We got complete saturation after 35 cycles. PCR products were separated on a 5% agarose gel, stained with ethidium bromide and visualized under UV light. Expected amplicon sizes were 246 bp (ERβ1), 141 bp (ERβ2), 91 bp (ERβ4) and 71 bp (ERβ5). Signal quantification during the exponential phase (30 cycles) showed that all isoforms are expressed in the 4 β clone with a slightly higher expression level for β5 isoform. (C) Growth rates of two distinct 4β expressing clones (4β#7 and 4β#22) were assessed by MTT assay at 24 h, 48 h, 72 h and 96 h post-seeding. Fold change in growth for each 4β clone was calculated relative to the corresponding control line at each time point, with the 24 h value set arbitrary to 1 as a control. No significant difference was observed between 4β#7 and 4β#22 clones as both exhibited reduced growth 48 h post-seeding that was maintained up to 96 h. Each experiment was repeated 5-7 times with quadruplicate measurements. Data represent means ± SEM. * $P \leq 0.05$, ** $P \leq 0.01$ vs. 24 h control, by the one-way ANOVA with Dunn's post hoc test. A.U., Arbitrary Units.

Supplementary Figure S5. Effect of transient expression of ERβ isoforms on HGrC1 cell proliferation. HGrC1 cells were transiently and efficiently (~70%) transfected by nucleofection with either Flag-Cherry (CTL), Flag-ERβ1(β1), Flag-ERβ2 (β2), Flag-ERβ (β4), or Flag-ERβ5 (β5). Cell proliferation was assessed by BrdU incorporation in Flag positive cells, detected by immunofluorescence. DAPI stains the nucleus. Magnification, ×20. Data represent means ± SEM from 4 replicates performed 4-8 times. ** $P \leq 0.01$, *** $P \leq 0.001$ vs. CTL, by the one-way ANOVA with Dunn's post hoc test.

Supplementary Figure S6. Analysis of Flag-ERβ isoform expression by western blotting. Western blot analysis was performed on HGrC1 cells transiently expressing Flag-mcherry control, Flag-ERβ1 (β1), Flag-β2 (β2), Flag-β4 (β4) or Flag-β5 (β5). Protein extracts were probed for Flag (upper panel) and GAPDH (lower panel). All ERβ isoforms were expressed at their expected molecular weights (59, 55, 54 or 53 kDa, respectively), as well as the flagged mcherry fusion protein (32 kDa).

Supplementary Figure S7. Analysis of differential gene expression in ERβ1-depleted hGCs, treated or not with E2. (A) Volcano plot showing differentially expressed genes (DEGs) in hGCs transduced with ERβ1 shRNA (B1), treated with either vehicle (CT) or E2. (B) Volcano plot showing DEGs between hGCs transduced with scramble (S) or ERβ1 (B1) shRNA, both treated with vehicle (CT). In both panels, the x-axis represents the log2 fold change (log2FC) in gene expression between groups, indicating up- or downregulation. The y axis shows the -log10(P-value), reflecting the statistical significance of differential expression. Genes significantly upregulated (log2FC > 1, $P < 0.05$) are shown in red; significantly 10 downregulated genes (log2FC < -1, $P < 0.05$) are shown in green; and non-significantly varying genes are displayed in blue. (C) List of DEGs between E2-treated hGCs transduced

with scramble (S) or ERβ1 (B1) shRNA in L1 and L3 samples (B1_E2 vs. S_E2). Genes highlighted in green are those found in common between the B1_E2 vs. S_E2 and S_E2 vs. S_CT comparisons (see Figure 6B).

Supplementary Table S1. Primer sequences for ERS2 variants integration in the MXS vector

Supplementary Table S2. The shRNA target sequences and primer sequences

Supplementary Table S3. Primer sequences for qPCR

Supplementary Table S4. Clinical characteristics of the women selected for the study

Supplementary Table S5. Upregulated genes in patient samples L1 and L3, compared to patient samples L2, L4 and L5 based on RNA-seq analysis. Cells derived for each patient and transduced with scramble shRNA (S) were treated with either vehicle (control, CT) or E2, and gene expression levels were measured as mapped read counts. Grey columns represent read counts in hGCs from patients L1 and L3, while blue columns show read counts from patients L2, L4, and L5. The yellow column displays the average expression ratio between the two patient groups (L1 and L3 vs. L2, L4, and L5) after CT or E2 treatment, and the white column reports the corresponding P-values from unpaired t-tests. Only genes that were significantly upregulated ($P \leq 0.05$) in both CT- and E2-treated cells from patients L1 and L3, compared to the other patient group, are included.

Supplementary Table S6. MCL-based protein clustering of ERβ1-specific partners in the vehicle-treated condition.

Supplementary Table S7. Functional annotation of MCL clusters of ERβ1-specific partners in the vehicle-treated condition.
