# Supplementary material for: Estrogen Receptor Beta Isoforms Interplay Integrates Nuclear and Extra‐Nuclear Signaling to Modulate Human Granulosa Cell Growth
Source: FASEB J. 2026 Oct 3;40(19):e72328. doi: 10.1096/fj.202602705RR (PMC13633505; doi:10.1096/fj.202602705RR)
Supplement: Supplementary file 1 [file FSB2-40-e72328-s001.zip › Supplemental Figures and Tables.pdf]

Estrogen receptor beta isoforms interplay integrates nuclear and extra-nuclear signaling to modulate human granulosa cell growth

Clémentine Marie, Inza-Noor Baskaran, Raphaël Corre, Anne Mayeur, Michaël Grynberg, Guillaume Chevreux, David L'Hôte, Joëlle Cohen-Tannoudji and Stéphanie Chauvin*

Supplementary Figures

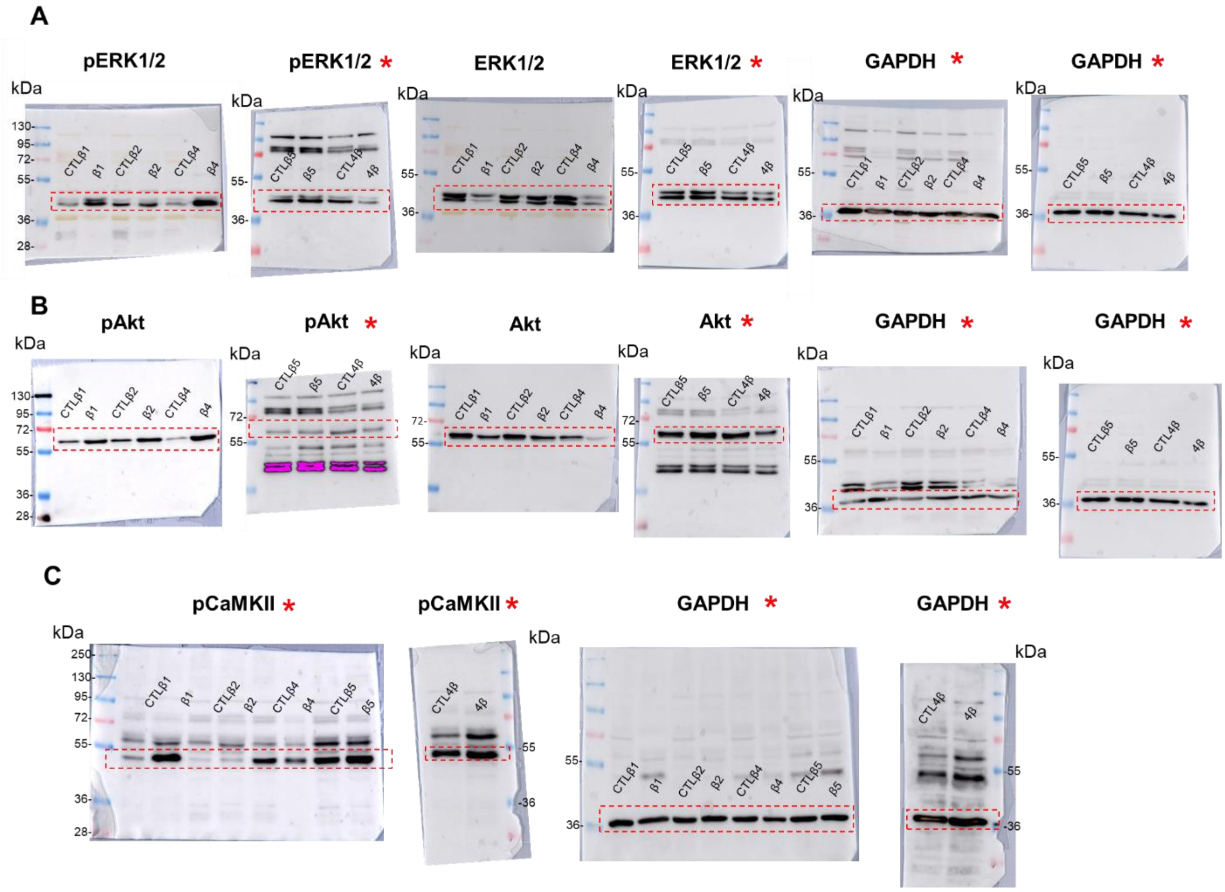

Supplementary Figure S1. Representative Western blots for phosphorylated ERK1/2, Akt and CaMKII, with corresponding controls in ERβ-expressing HGrC1 cells. Proteins extracted from each β-expressing HGrC1 line (*ESR2* variants v1 (β1), v2 (β2), v4 (β4), v5 (β5) or all four isoforms (4β)), along with their respective control (CTL), were analysed by Western blot. Blots were probed to detect: (a) phosphorylated and total ERK1/2 (P-ERK and ERK, 42-44 kDa), (b) phosphorylated and total Akt (P-Akt and Akt, 60 kDa), and (c) phosphorylated CaMKII (P-CaMKII, 47 kDa) and GAPDH (37 kDa). The images are representative of at least 4 independent experiments. Blots marked with a red asterisk (*) indicate membranes previously probed with other antibodies; residual signals are visible but do not represent non-specific bands.

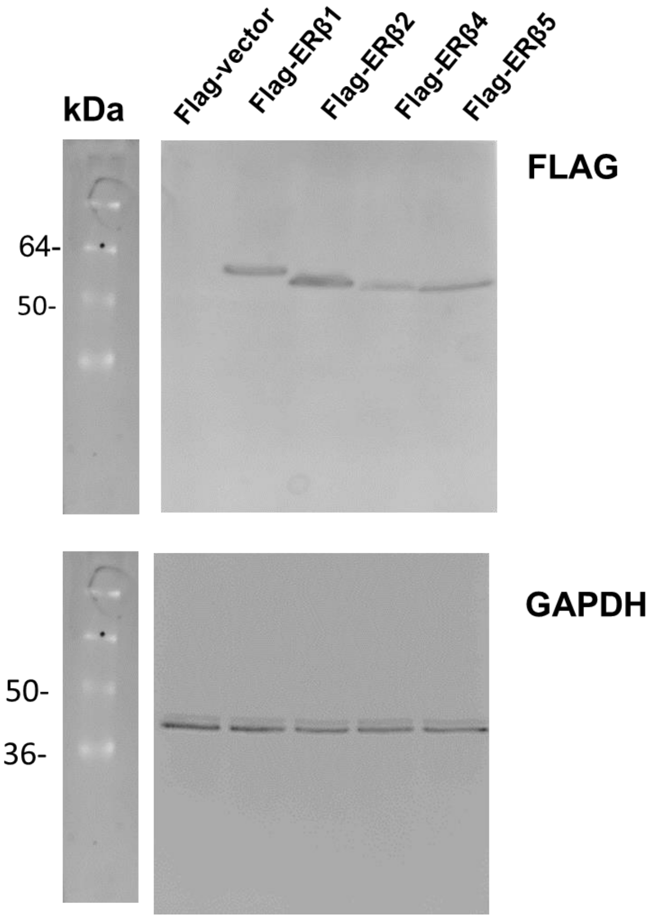

Supplementary Figure S2. Expression of Flag-tagged ERβ isoforms in HGrC1 cells before immunoprecipitation. HGrC1 were transiently transfected with either Flag-control, Flag-ERβ1, Flag-ERβ2, Flag-ERβ4 or Flag-ERβ5 for 48 h. Protein extracts were prepared as described in the Methods section. An aliquot (40 µg) of each protein extract was used in Western blot analysis to detect Flag-tagged proteins and GAPDH. ERβ1, ERβ2, ERβ4 and ERβ5 were detected at the expected molecular weights of 59, 55, 54 and 53 kDa respectively.

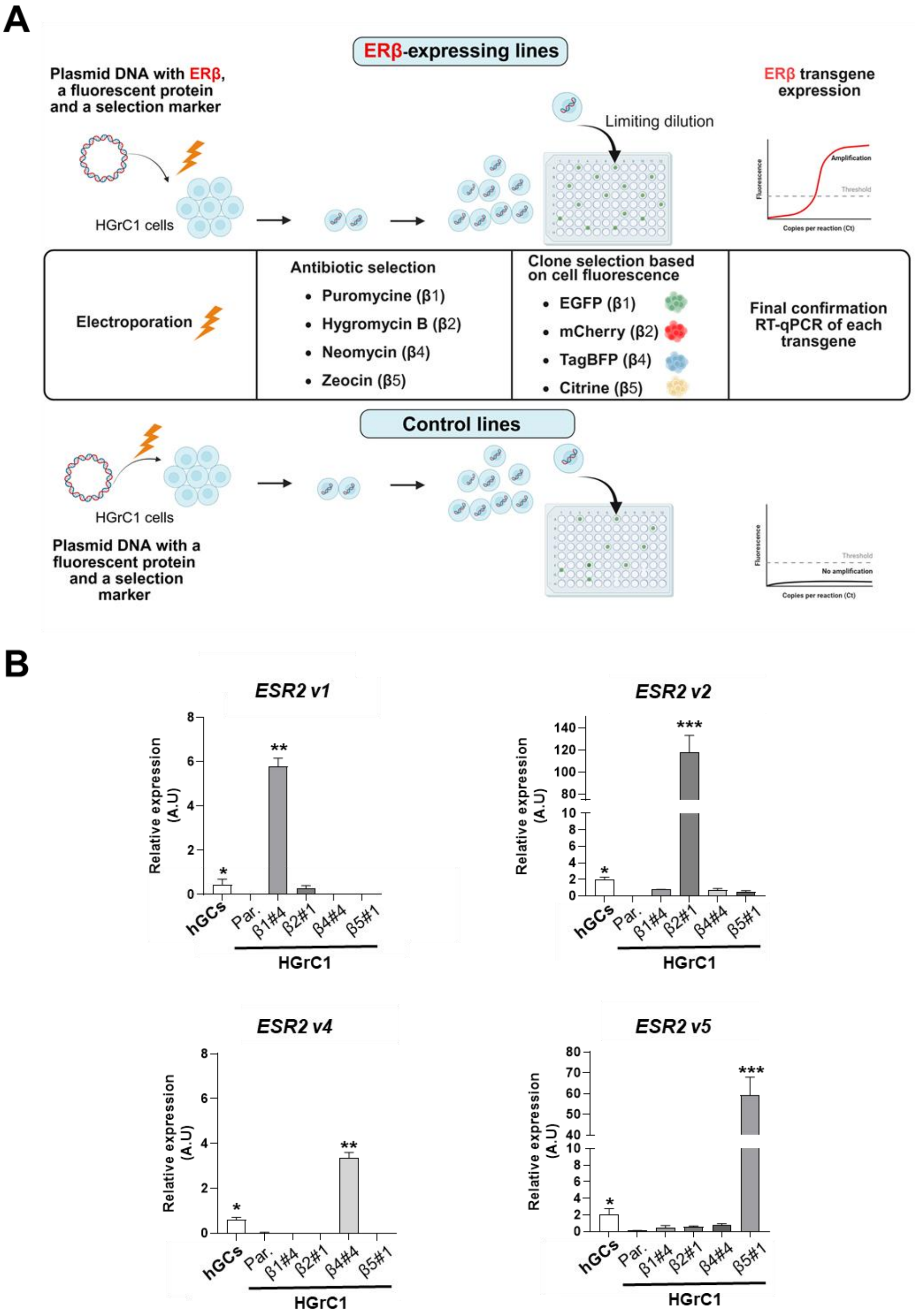

Supplementary Figure S3. **Generation and validation of HGrC1 clonal cell Lines expressing a single ERβ isoform**. (A) Protocol of establishment of HGrC1 clonal cell lines stably expressing individual ERβ isoforms (*ESR2* variants v1 (β1), v2 (β2), v4 (β4) or v5 (β5)). Each isoform was associated with a specific fluorescent protein (EGFP, mCherry, TagBFP and Citrine for β1, β2, β4 and β5 respectively) and a corresponding antibiotic resistant marker (puromycin, hygromycin B, neomycin and zeocin, respectively). Control cell lines expressed only the respective fluorescent proteins alongside with the matching selection marker. Following electroporation, cells were selected using the corresponding antibiotic for 4-6 weeks. Monoclonal lines were then generated by limiting dilution, and positives clones were identified based on fluorescence. After clone expansion, transgene expression, as well as endogenous *ESR2* isoform expression, were confirmed by RT-qPCR as described in the

Methods. Created in BioRender. (B) Total mRNA was extracted from β1#4-, β2#1-, β4#4- and β5#1-expressing clones, along with their corresponding control cell lines (CTL). Expression levels of transgenic *ESR2* were quantified by RT-qPCR and normalized to *ACTB*. Data are shown as means ± SEM from duplicates across 3 independent experiments.

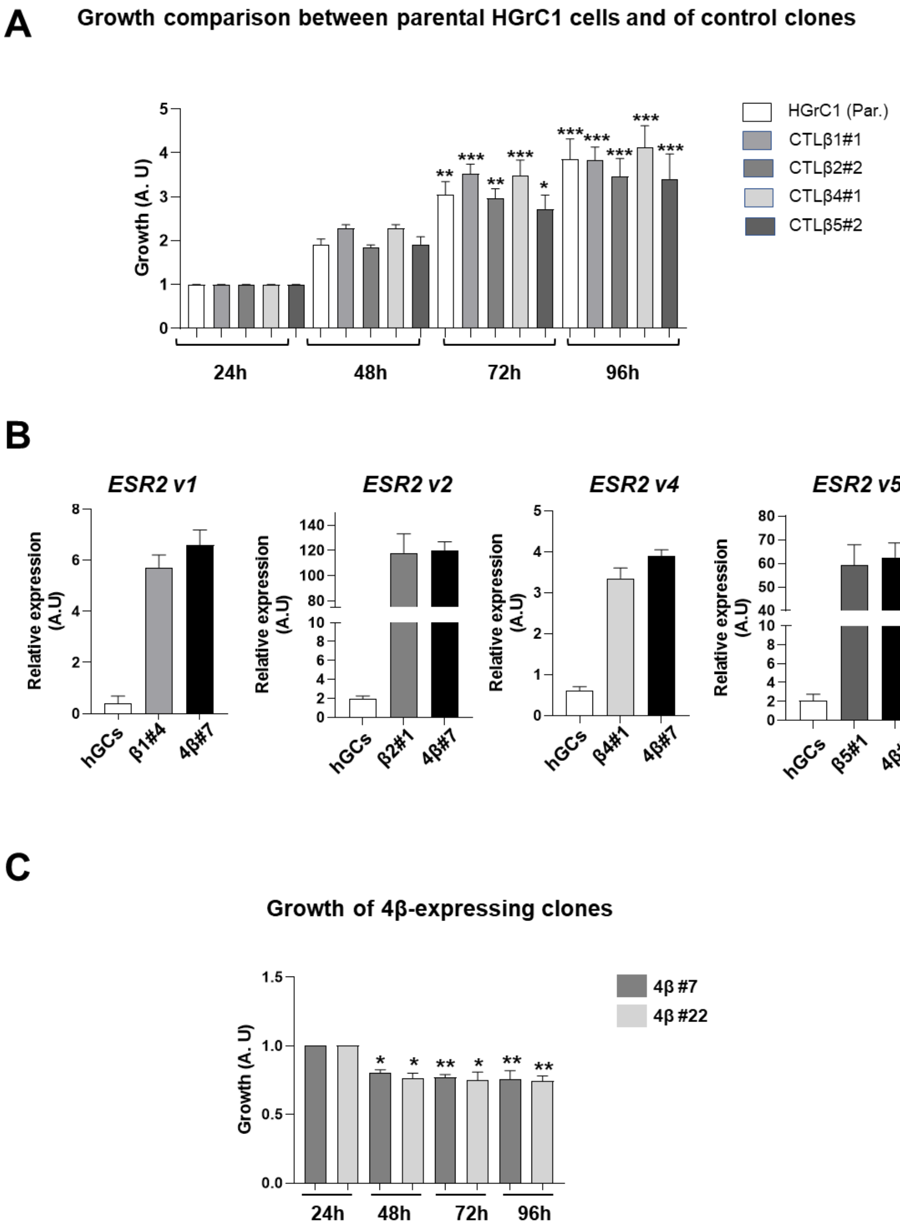

Supplementary Figure S4. Growth rate analysis and ESR2 variant expression in clonal cell lines. (A) Growth rate comparison between parental HGrC1 cells and control clones. The growth rates of parental (Par.) HGrC1 cells and clonal lines expressing only the fluorescent reporter proteins (CTLβ1#1, CTLβ2#2, CTLβ4#1 and CTLβ5#2) were assessed over 96 h. Formazan production, directly proportional to the number of viable cells, was measured by recording changes in absorbance at 575 nm. Fold changes in growth were calculated as the ratio of the OD_{575nm} values at 48 h, 72h and 96 h relative to 24 h, with 24 h-value set to 1 as a control. Of note, the number of cells was similar (DO_{575} ~ 0.6) for each cell line at 24 h. No significant differences in growth were observed. This indicates that the stable line engineering procedure did not affect HGrC1 cell growth. Data are representative of 4 independent experiments performed in quadruplicate. Data are presented as means ± SEM. * $P \leq 0.05$, ** $P \leq 0.01$, *** $P \leq 0.001$ vs. 24 h control, by ordinary one-way ANOVA. A.U., Arbitrary

Units. (B) Total RNA was extracted from the four single β-expressing clones (β1#4, β2#1, β4#1 and β5#4), the 4β#7-expressing clone and their respective control cell line (CTL). Expression levels of transgenic *ESR2* variants (in β-expressing clones) were quantified by semi-quantitative RT-PCR. PCR aliquots (5 µL) were collected at 20, 25 and 30 cycles. We got complete saturation after 35 cycles. PCR products were separated on a 5% agarose gel, stained with ethidium bromide and visualized under UV light. Expected amplicon sizes were 246 bp (ERβ1), 141 bp (ERβ2), 91 bp (ERβ4) and 71 bp (ERβ5). Signal quantification during the exponential phase (30 cycles) showed that all isoforms are expressed in the 4 β clone with a slightly higher expression level for β5 isoform. (C) Growth rates of two distinct 4β-expressing clones (4β#7 and 4β#22) were assessed by MTT assay at 24 h, 48 h, 72 h and 96 h post-seeding. Fold change in growth for each 4β clone was calculated relative to the corresponding control line at each time point, with the 24 h value set arbitrary to 1 as a control. No significant difference was observed between 4β#7 and 4β#22 clones as both exhibited reduced growth 48 h post-seeding that was maintained up to 96 h. Each experiment was repeated 5-7 times with quadruplicate measurements. Data represent means ± SEM. * $P \leq 0.05$, ** $P \leq 0.01$ vs. 24 h control, by the one-way ANOVA with Dunn's post hoc test. A.U., Arbitrary Units.

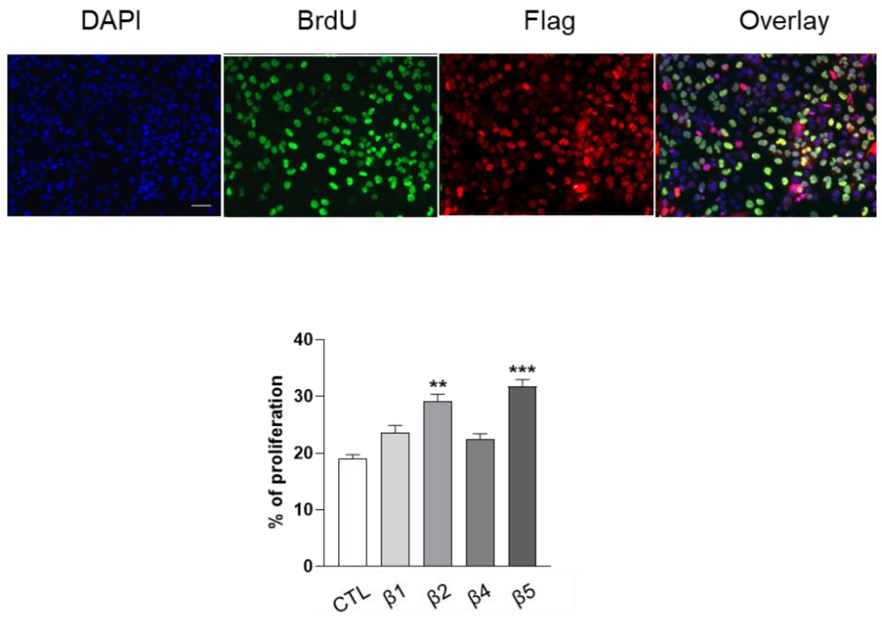

Supplementary Figure S5. Effect of transient expression of ERβ isoforms on HGrC1 cell proliferation. HGrC1 cells were transiently and efficiently (~70%) transfected by nucleofection with either Flag-Cherry (CTL), Flag-ERβ1(β1), Flag-ERβ2 (β2), Flag-ERβ (β4), or Flag-ERβ5 (β5). Cell proliferation was assessed by BrdU incorporation in Flag-positive cells, detected by immunofluorescence. DAPI stains the nucleus. Magnification, ×20. Data represent means ± SEM from 4 replicates performed 4-8 times. ** $P \leq 0.01$, *** $P \leq 0.001$ vs. CTL, by the one-way ANOVA with Dunn's post hoc test.

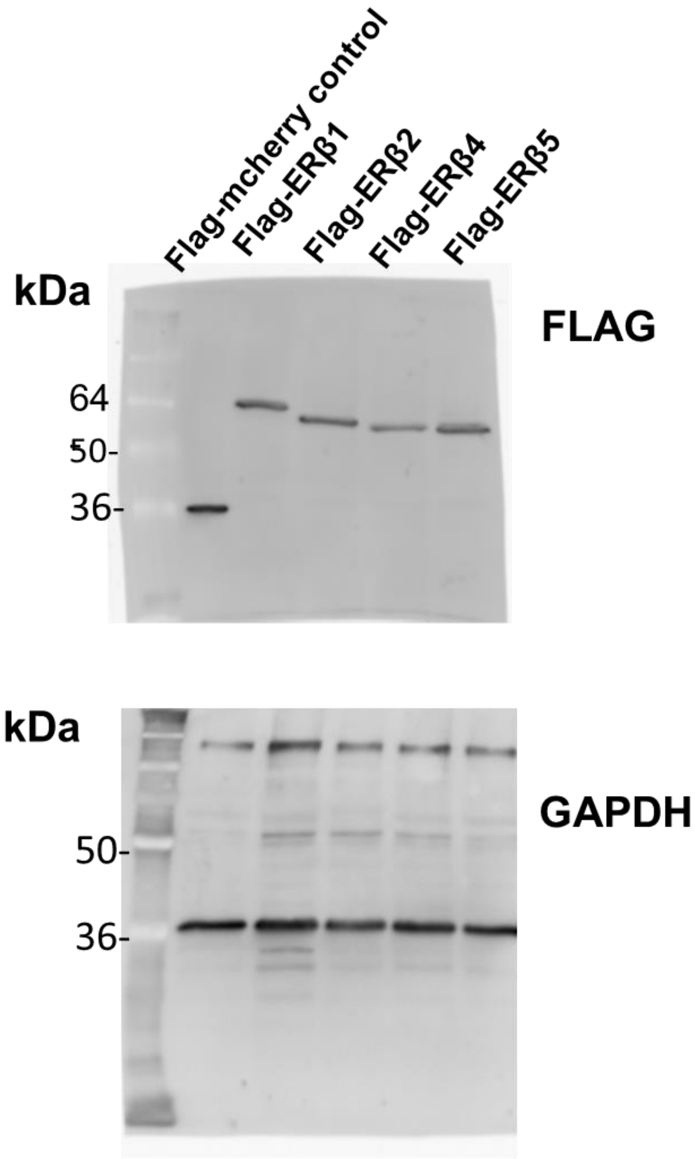

Supplementary Figure S6. Analysis of Flag-ERβ isoform expression by western blotting. Western blot analysis was performed on HGrC1 cells transiently expressing Flag-mcherry control, Flag-ERβ1 (β1), Flag-β2 (β2), Flag-β4 (β4) or Flag-β5 (β5). Protein extracts were probed for Flag (upper panel) and GAPDH (lower panel). All ERβ isoforms were expressed at their expected molecular weights (59, 55, 54 or 53 kDa, respectively), as well as the flagged mcherry fusion protein (32 kDa).

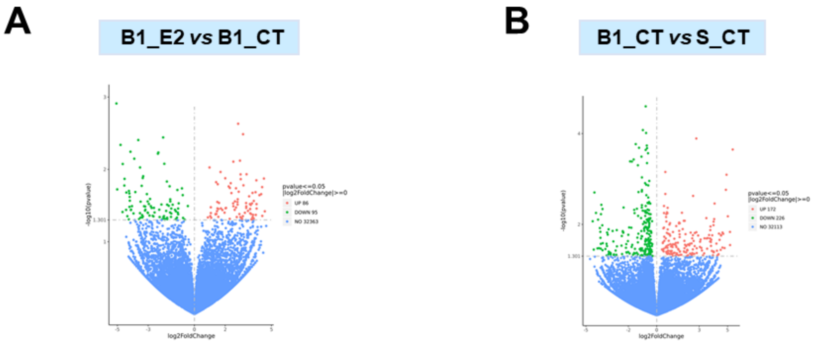

C

gene_id	log2FoldCha	pvalue	padj	gene_name
ENSG000001	-23.5895058	1.07445560	1.77252941	CCDC141
ENSG000001	-22.9591234	9.20128063	7.58967633	ADGRF2
ENSG000001	-24.0211645	2.08213339	1.14496515	MYT1L
ENSG000001	-23.4406692	9.64486456	2.30557241	PAX4
ENSG000001	-23.4179506	1.02343040	2.30557241	ADGRA1
ENSG000001	-23.3547559	1.20665702	2.30557241	ST8SIA3
ENSG000001	-23.3239142	1.30741152	2.30557241	CLNK
ENSG000000	-23.2936779	1.41425848	2.30557241	CACNA1S
ENSG000002	-23.2684299	1.51001861	2.30557241	LINC01289
ENSG000001	-23.2644465	1.52570082	2.30557241	ENAM
ENSG000002	-23.2615346	1.53732778	2.30557241	LINC01488
ENSG000002	-23.1408220	2.10090005	2.81863771	AL031593.1
ENSG000002	-23.1093934	2.27828380	2.81863771	MROH5
ENSG000002	-23.0737070	2.49774494	2.81863771	POTEKP
ENSG000002	-23.0555039	2.61755573	2.81863771	KCNU1
ENSG000002	-23.0386172	2.73372149	2.81863771	KARSP3
ENSG000002	-22.9835565	3.14900781	3.05583423	AC079062.1
ENSG000001	-22.9042727	3.85736297	3.53527316	CTCFL
ENSG000001	-22.8278297	4.69009149	3.99553069	PLPPR1
ENSG000001	-22.8151602	4.84394823	3.99553069	BPIFB4
ENSG000002	-22.7416539	5.84021876	4.58777900	LINC01247
ENSG000001	-22.7020832	6.45837090	4.58777900	ELAVL4
ENSG000001	-22.7011470	6.47352881	4.58777900	OR4N2
ENSG000002	-22.6891235	6.67434660	4.58777900	LINC02538
ENSG000001	-22.6327379	7.69944212	4.94318427	ITPRID1
ENSG000002	-22.6221703	7.90784693	4.94318427	DUXB
ENSG000002	-22.6131407	8.09031795	4.94318427	AC073324.1
ENSG000002	-22.5968175	8.43132879	4.96755825	THRAP3P1
ENSG000002	-22.5456092	9.59608463	5.33209070	AC010307.3
ENSG000002	-22.5295791	9.99149578	5.33209070	AL161757.4
ENSG000000	-22.5284309	1.00196891	5.33209070	MYH13
ENSG000001	-22.5005912	1.07493349	5.48183725	RBPJL
ENSG000001	-22.4804998	1.13069074	5.48183725	OR7A15P
ENSG000002	-22.4673322	1.16879747	5.48183725	PCDHB19P
ENSG000002	-22.4595849	1.18981039	5.48183725	FAM99B
ENSG000002	-22.4580331	1.19625471	5.48183725	LINC02474
ENSG000000	-22.4376229	1.25871891	5.61218538	OR1I1
ENSG000001	-22.4254638	1.29845689	5.63701143	CYP2A13
ENSG000001	-22.3960863	1.39769816	5.91226324	TERT
ENSG000001	-22.3668864	1.50392427	6.20255968	C10orf120
ENSG000001	-22.2836562	1.85212240	7.38006947	CSH1
ENSG000002	-22.2702596	1.91530091	7.38006947	AL662791.2
ENSG000002	-22.2684751	1.92364058	7.38006947	AC005005.4
ENSG000002	-22.2139423	2.20383499	8.26287861	HNRNPA1P5
ENSG000002	-22.1138536	2.81206369	1.03090254	AC005906.2
ENSG000002	-22.0932363	2.96190756	1.06223019	AL365338.1
ENSG000002	-22.0462541	3.25958634	1.14411480	KRT16P6
ENSG000002	-20.8610575	5.59382009	1.92252604	AP003900.1
ENSG000002	-20.8084483	6.33479072	2.13275597	OR7E26P
ENSG000002	-20.4206361	1.53977072	5.08031952	TOMM22P4

B1_E2 *vs* S_E2

L1 and L3 samples only

Supplementary Figure S7. Analysis of differential gene expression in ERβ1-depleted hGCs, treated or not with E2. (A) Volcano plot showing differentially expressed genes (DEGs) in hGCs transduced with ERβ1 shRNA (B1), treated with either vehicle (CT) or E2. (B) Volcano plot showing DEGs between hGCs transduced with scramble (S) or ERβ1 (B1) shRNA, both treated with vehicle (CT). In both panels, the x-axis represents the $\log_2$ fold change ($\log_2$FC) in gene expression between groups, indicating up- or downregulation. The y-axis shows the $-\log_{10}$(P-value), reflecting the statistical significance of differential expression. Genes significantly upregulated ($\log_2$FC > 1, $P < 0.05$) are shown in red; significantly

downregulated genes ($log_2FC < -1$, $P < 0.05$) are shown in green; and non-significantly varying genes are displayed in blue. (C) List of DEGs between E2-treated hGCs transduced with scramble (S) or ERβ1 (B1) shRNA in L1 and L3 samples (B1_E2 vs. S_E2). Genes highlighted in green are those found in common between the B1_E2 vs. S_E2 and S_E2 vs. S_CT comparisons (see Figure 5D).

Supplementary Tables

Supplementary Table S1. Primer sequences for ERS2 variants integration in the MXS vector

Gene	Forward (5'→3')	Reverse (5'→3')
ESR2 v1	AATACGCGTAACTCGAGCCGCCACCATGGATATAAAAAACTCACC	CTAGTCGACCTGAGACTGTGGGTTCTG
ESR2 v2	AATACGCGTAACTCGAGCCGCCACCATGGATATAAAAAACTCACC	CTAGTCGACCTGCTCCATCGTTGCTTCAG
ESR2 v4	AATACGCGTAACTCGAGCCGCCACCATGGATATAAAAAACTCACC	CTAGTCGACAGATAACTTCAAAATGAATGAATTGC
ESR2 v5	AATACGCGTAACTCGAGCCGCCACCATGGATATAAAAAACTCACC	CTAGTCGACGGGCGCGTACCTCGCATG

MluI, *XhoI* and *SalI*, specific sequences are highlighted in green, blue and red respectively

Supplementary Table S2: The shRNA target sequences and primer sequences

shRNA	Target sequence (5'→3')	shRNA sequence Forward (5'→3')	shRNA sequence reverse (5'→3')
ERβ1	GGAACATCTGCTCAACATGAA	*CCGGT*GGAACATCTGCTCAACATGAA**CTCGAG**TTCATGTTGAGCAGATGTTCC TTTTT *G*	*AATTC*AAAAAGGAACATCTGCTCAACATGAA**CTCGAG**TTCATGTTGAGCAGATGTTCC*A*
Scramble	GAAACAATGCGGACCAATTCT	*CCGGT*GAAACAATGCGGACCAATTCT**CTCGAG** AGAATTGGTCCGCATTGTTTC TTTTT *G*	*AATTC*AAAAAGAAACAATGCGGACCAATTCT**CTCGAG**AGAATTGGTCCGCATTGTTTC*A*

Specific sequence in yellow, bold loop, restriction sequence in *italics*

Supplementary Table S3. Primer sequences for qPCR

**Genes**	**Forward (5’→3’)**	**Reverse (5’→3’)**	**Amplicon size (bp)**
EGR1	GAGCAGCCCTACGAGCAC	GTCTCCACCAGCACCTTCTC	83 Efficiency = 102%
CTNNB1	GAGCCTGCCATCTGTGCTCT	ACGCAAAGGTGCATGATTTG	215 Efficiency = 89.5%
MYC	CAGCTGCTTAGACGCTGGATT	GTAGAAATACGGCTGCACCGA	136 Efficiency = 97.6%
ENAM	CAATGCACATGCCCCGAATG	GACCGTTTCCAAAGAAGGGC	123 Efficiency = 91.7%
CACNA1A	GAATTGCATCGTCCTCGCAC	TGGTTCTGTGTCATCCAGCC	85 Efficiency = 90.5%
GRIK4	GAACTGAACCGGAGCCAAGA	CAATGTGGCCGGTAAGACCT	130 Efficiency = 98.8%
ANO2	TTATTCTGTCCGCCCGTCTG	GATGTCCGTGGGGATGTCTG	104 Efficiency = 90.5%
BAX	ATGTTTTCTGACGGCAACTTC	AGTCCAATGTCCAGCCCAT	139 Efficiency = 98.8%
BCL2	AACGTGCCTCATGAAATAAAG	TTATTGGATGTGCTTTGCATTC	148 Efficiency = 104.8%
ACTB	GTGAAGGTGACAGCAGTCGGTT	GAAGTGGGGTGGCTTTTAGGA	165 Efficiency = 97%
GAPDH	GACCCCTTCATTGACCTCAAC	CTTCTCCATGGTGGTGAAGA	227 Efficiency = 98.5%
ESR2 v1	GTCAGGCATGCGAGTAACAA	GGGAGCCCTCTTTGCTTTTA	192 Efficiency = 90.9%
ESR2 v2	TCTCCTCCCAGCAGCAATCC	GGTCACTGCTCCATCGTTGC	162

			Efficiency = 96%
ESR2 v4	GTGACCGATGCTTTGGTTTG	ATCTTTCATTGCCCACATGC	210 Efficiency = 96%
ESR2 v5	GATGCTTTGGTTTGGGTGAT	CCTCCGTGGAGCACATAATC	177 Efficiency = 95%

Supplementary Table S4. Clinical characteristics of the women selected for the study

# women	Age (y)	BMI (kg/m^2)	Serum FSH (UI/L)	Serum LH (UI/L)	AMH (ng/mL)	E2 (pg/mL)	AFC
L1	35	18.8	7.6	7.9	5.6	65	36
L2	36	21.4	8.5	4.8	1.0	44	16
L3	36	26.2	8	4.9	1.52	52	10
L4	36	22.0	5.4	5.0	3.4	29	18
L5	37	20.2	4.2	7.9	1.3	135	13

Abbreviations: BMI, body mass index; LH, luteinizing hormone; FSH, Follicle-stimulating hormone; AMH, anti-Müllerian hormone; AFC, antral follicle count; E2, 17β-estradiol.

Supplementary Table S5: Upregulated genes in patient samples L1 and L3, compared to patient samples L2, L4 and L5 based on RNA-seq analysis.

Cells derived for each patient and transduced with scramble shRNA (S) were treated with either vehicle (control, CT) or E2, and gene expression levels were measured as mapped read counts. Grey columns represent read counts in hGCs from patients L1 and L3, while blue columns show read counts from patients L2, L4, and L5. The yellow column displays the average expression ratio between the two patient groups (L1 and L3 vs. L2, L4, and L5) after CT or E2 treatment, and the white column reports the corresponding *P*-values from unpaired t-tests. Only genes that were significantly upregulated ($P \leq 0.05$) in both CT- and E2-treated cells from patients L1 and L3, compared to the other patient group, are included.

gene_id	L1_S_CT	L3_S_CT	L2_S_CT	L4_S_CT	L5_S_CT	*P*-values	Mean ratio	L1_S_E2	L3_S_E2	L2_S_E2	L4_S_E2	L5_S_E2	*P*-Values	Mean ratio	gene_name
ENSG00000161011	31226.38	32447.45	6049.87	5430.66	12869.63	0.0070	3.92	62467.98	60687.09	6093.68	5625.42	13251.48	0.0008	7.40	SQSTM1
ENSG00000260428	130.05	146.87	54.63	25.73	54.37	0.0061	3.08	469.29	536.51	51.86	38.50	64.92	0.0380	9.71	SCX
ENSG00000143815	1118.75	1106.55	262.69	439.43	445.56	0.0062	2.91	1114.93	1094.98	272.27	392.12	477.32	0.0056	2.90	LBR
ENSG00000181896	434.026	443.28	160.67	183.18	228.75	0.0047	2.30	1329.17	1363.24	190.77	179.34	199.34	0.0039	7.09	ZNF101
ENSG00000146242	428.54	449.93	162.27	224.35	226.10	0.0029	2.15	803.04	663.33	125.02	273.57	262.43	0.0280	3.33	TPBG
ENSG00000116922	515.50	531.01	218.50	217.14	305.00	0.0076	2.12	699.56	824.28	273.20	212.78	337.41	0.0323	2.78	C1orf109
ENSG00000198909	1055.29	974.96	469.14	458.99	586.13	0.0045	2.01	2589.84	2458.21	418.59	458.99	640.08	0.0004	4.99	MAP3K3
ENSG00000181220	1353.00	1263.40	716.57	525.88	719.40	0.0036	2.00	2846.34	2392.37	718.65	475.20	677.57	0.0499	4.20	ZNF746
ENSG00000116954	1050.59	1153.07	245.01	248.02	566.24	0.0104	3.12	789.92	860.86	273.20	293.83	582.47	0.0366	2.15	RRAGC
ENSG00000174197	1953.12	1776.46	797.71	614.38	1139.10	0.0116	2.19	1702.27	1814.39	853.86	434.67	1032.36	0.0231	2.27	MGA
ENSG00000131979	429.32	492.46	72.30	161.57	242.67	0.0143	2.90	770.97	665.76	88.90	195.55	262.43	0.0084	3.94	GCH1
ENSG00000176170	2105.10	1931.31	430.58	612.32	1124.52	0.0152	2.79	12510.50	11239.98	432.48	681.90	1124.71	0.0224	15.91	SPHK1
ENSG00000165244	47.79	57.82	5.62	4.12	25.20	0.0165	4.53	94.73	87.79	13.89	14.18	21.94	0.0025	5.47	ZNF367
ENSG00000121039	1262.12	1369.07	487.62	237.73	711.44	0.0166	2.75	2951.28	2948.39	551.02	226.96	694.94	0.0031	6.01	RDH10
ENSG00000204839	176.27	208.02	62.66	28.81	62.99	0.0182	3.73	890.48	897.44	50.01	28.37	64.92	0.00004	18.71	MROH6
ENSG00000182118	94.01	108.33	23.30	9.26	54.37	0.0197	3.49	260.88	338.98	6.48	20.26	72.24	0.0465	9.09	FAM89A
ENSG00000164309	334.53	283.78	41.77	53.51	170.40	0.0202	3.49	311.89	417.02	30.56	47.62	174.65	0.0416	4.32	CMYA5
ENSG00000143367	537.44	627.38	282.77	185.24	334.17	0.0210	2.18	686.44	670.64	282.46	168.19	365.76	0.0178	2.49	TUFT1
ENSG00000162032	27.42	31.90	16.87	5.14	12.60	0.0216	2.57	64.13	80.48	8.33	1.01	22.86	0.0218	6.73	SPSB3
ENSG00000225648	611.08	631.36	179.14	228.46	373.29	0.0224	2.39	1068.29	1085.22	162.99	271.54	373.99	0.0049	4.00	SBDSP1
ENSG00000135378	379.18	312.36	84.35	58.66	143.22	0.0236	3.62	616.49	629.18	84.27	74.98	204.82	0.0060	5.13	PRRG4
ENSG00000168003	29653.23	32841.56	3541.88	5581.94	17422.73	0.0253	3.53	107175.82	108897.78	3677.51	6615.34	18340.11	0.0015	11.32	SLC3A2
ENSG00000121068	89.31	113.64	8.03	25.73	43.76	0.0302	3.93	620.86	682.84	1.85	34.50	47.55	0.0123	23.32	TBX2
ENSG00000115339	154.34	184.09	41.77	84.39	110.06	0.0359	2.15	183.63	221.92	37.97	77.00	88.70	0.0245	2.99	GALNT3
ENSG00000013810	432.46	356.89	175.93	161.57	96.80	0.0369	2.73	746.20	729.17	149.10	173.26	107.90	0.0002	5.14	TACC3
ENSG00000167703	363.52	437.30	116.48	216.11	258.59	0.0387	2.03	734.54	816.96	119.46	236.08	296.26	0.0037	3.57	SLC43A2
ENSG00000137310	137.12	110.99	36.95	46.31	64.98	0.049	2.51	107.85	102.42	38.89	28.37	69.49	0.0353	2.31	TCF19
